# Molecular Epidemiological Evidence Implicates Cattle as a Primary Reservoir of *Campylobacter jejuni* Infecting People via Contaminated Chickens

**DOI:** 10.3390/pathogens11111366

**Published:** 2022-11-16

**Authors:** Januana S. Teixeira, Valerie F. Boras, Benjamin M. Hetman, Eduardo N. Taboada, G. Douglas Inglis

**Affiliations:** 1 Lethbridge Research and Development Centre, Agriculture and Agri-Food Canada, Lethbridge, AB T1J 4B1, Canada; 2 Chinook Regional Hospital, Alberta Health Services, Lethbridge, AB T1J 1W5, Canada; 3 Office of Public Health Field Services and Training, Public Health Agency of Canada, Winnipeg, MB R3C 0P4, Canada; 4 National Microbiology Laboratory, Public Health Agency of Canada, Winnipeg, MB R3E 3R2, Canada

**Keywords:** subtypes, comparative genomic fingerprinting, CGF40, source attribution, one health, public health

## Abstract

The study aimed to determine the relative contribution of cattle to the burden of illness in a model agroecosystem with high rates of human campylobacteriosis (≥ 115 cases/100 K), and high densities of cattle, including large numbers of cattle housed in confined feeding operations (i.e., in southwestern Alberta, Canada). To accomplish this, a large-scale molecular epidemiological analysis of *Campylobacter jejuni* circulating within the study location was completed. In excess of 8000 isolates of *C. jejuni* from people (*n* = 2548 isolates), chickens (*n* = 1849 isolates), cattle (*n* = 2921 isolates), and water (*n* = 771 isolates) were subtyped. In contrast to previous studies, the source attribution estimates of clinical cases attributable to cattle vastly exceeded those attributed to chicken (i.e., three- to six-fold). Moreover, cattle were often colonized by *C. jejuni* (51%) and shed the bacterium in their feces. A large proportion of study isolates were found in subtypes primarily associated with cattle (46%), including subtypes infecting people and those associated with chickens (19%). The implication of cattle as a primary amplifying reservoir of *C. jejuni* subtypes in circulation in the study location is supported by the strong cattle association with subtypes that were found in chickens and in people, a lack of evidence indicating the foodborne transmission of *C. jejuni* from beef and dairy, and the large number of cattle and the substantial quantities of untreated manure containing *C. jejuni* cells. Importantly, the evidence implicated cattle as a source of *C. jejuni* infecting people through a transmission pathway from cattle to people via the consumption of chicken. This has implications for reducing the burden of campylobacteriosis in the study location and elsewhere.

## 1. Introduction

Campylobacteriosis is one of the most common foodborne bacterial-incited diseases of human beings in many countries including Canada [1,2,3]. *Campylobacter jejuni* is a primary incitant of campylobacteriosis, and this bacterium is responsible for the majority of cases globally (≈95%) [4]. *Campylobacter jejuni* colonizes a large number of hosts, of which many do not show any overt evidence of being infected [5]. Furthermore, the bacterium is readily isolated from environmental sources such as feces and surface waters [6]. Chickens are a chief reservoir of *C. jejuni* infecting people, and the consumption of inadequately prepared chicken meat contaminated with the bacterium is generally considered to be the primary contributor to campylobacteriosis [7,8]. However, some aspects of the epidemiology of campylobacteriosis are not fully resolved at present and remain as significant knowledge gaps, including the extent to which other sources contribute to disease, and additional mechanisms by which the bacterium is transmitted to people. Emerging evidence indicates that not all strains of *C. jejuni* represent an equivalent risk to people [9,10,11], and examining the bacterium at a subspecies level of resolution is expected to aid in the identification of the primary reservoirs of high-risk strains and the relevant transmission mechanisms (i.e., the transmission of high-risk strains).

Southwestern Alberta (SWA), Canada, is a location that is characterized by high rates of campylobacteriosis (≥ 115 infections/100 K) along with high densities of livestock [1]. Our research has consistently indicated that *C. jejuni* strains observed in people in SWA are strongly associated with the subtypes linked to cattle, including strains resistant to key antibiotics [1,12,13]. However, the role of cattle in the epidemiology of campylobacteriosis in SWA is not completely understood. Our research has shown that the consumption of water and beef does not contribute significantly to disease in the region [13,14,15]. Thus, although *C. jejuni* subtypes originating from the cattle reservoir are prevalent in people in SWA, foodborne or waterborne routes do not appear to be significant sources of direct exposure of people to high-risk subtypes of the bacterium from cattle.

Source attribution, which aims to quantify the proportion of cases of disease linked to potential pathogen sources, can provide insights on the relative importance of various sources to the burden of illness [16]. We reasoned that a large-scale molecular epidemiological investigation using SWA as a model agroecosystem in which high densities of cattle are a dominant feature, could shed light onto the possible role of cattle in the epidemiology of campylobacteriosis. We hypothesized that in SWA source attribution based on the isolation and molecular characterization of isolates from multiple sources would reveal a higher proportion of campylobacteriosis cases attributable to cattle than observed elsewhere. To test this hypothesis we established the following objectives: (i) obtain *C. jejuni* isolates from the cattle, chickens, human beings, and water in SWA; (ii) subtype a large number of the isolates (>8000) using comparative genomic fingerprinting (CGF40), a high throughput, and a high-resolution subtyping method; (iii) comparatively examine the *C. jejuni* subtypes recovered from the four sources in SWA to identify high-risk strains (i.e., clinically relevant strains) that are circulating in the region and reservoirs from which they may have originated; and (iv) generate source attribution estimates for the proportion of human campylobacteriosis cases attributable to the cattle, chickens, and water in SWA.

## 2. Results

### 2.1. Characteristics of the Study Area

**Figure 1:**
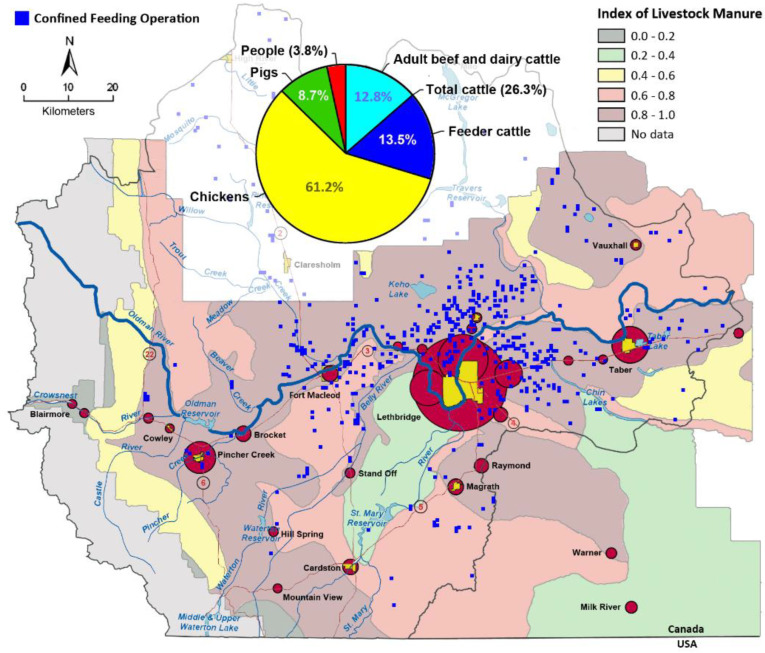
Animal densities, livestock manure production, locations of confined feeding operations (CFOs) in southwestern Alberta (SWA), and distribution of infections by *Campylobacter jejuni* (i.e., the size of red circles is proportional to the number of annual infections). Sources used were the 2011 Census of Agriculture for Alberta [17], 2011 Municipal Affairs Population List for Alberta [18], the Agriculture Land Resource Atlas of Alberta [19], and Inglis et al. [1]. The Oldman River watershed map with the location of CFOs within the watershed was provided courtesy of the Oldman Watershed Council. The boundary indicated on the map represents the Oldman River watershed. The area denoted by the manure index colors represents the boundaries of the Chinook Health Region of SWA.

Southwestern Alberta is a region of mixed agriculture, and it represents a gradation of agricultural intensity from west (mountains) to east (cultivated short grass prairie) and north to south based on the availability of irrigation. The region has a high density of livestock production with ≈2.7 M chickens, ≈0.39 M pigs, and ≈1.1 M cattle, with the highest densities of livestock within Lethbridge County (Supplemental Figure S1A). The majority of the cattle in SWA (51%) at any given time are housed in confined feeding operations (CFOs), with a conspicuously high number in CFOs concentrated in Lethbridge County (Figure 1). The rates of campylobacteriosis in SWA are consistently higher than both the provincial and national averages, with an average of 67.9 infections/100 K (range of 42.7 to 94.0 infections/100 K) (Supplemental Figure S2A). Moreover, the highest rates of campylobacteriosis in Alberta consistently occur in SWA (i.e., the Chinook Health Region) (Supplemental Figure S2B). Campylobacteriosis in SWA is primarily the result of infections by *C. jejuni*, and infections occur throughout the year, with peaks in late summer and early autumn (Supplemental Figure S3). The majority of cases of infection by *C. jejuni* occur in people living in Lethbridge and the surrounding communities (Figure 1).

### 2.2. A High Degree of Campylobacter jejuni Strain Diversity Was Observed

A total of 8089 *C. jejuni* isolates collected between 2004 and 2018 from people (*n* = 2548 isolates), cattle (*n* = 2921 isolates), chickens (*n* = 1849 isolates), and water (*n* = 771 isolates) in SWA were subtyped by CGF40 (Supplemental Table S1), a subtyping method that targets the presence or absence of 40 accessory loci. Overall, 1712 distinct CGF40 clusters were observed at a 95% level of resolution (Supplemental Table S2), which included 1064 clusters composed of a single isolate (i.e., singletons), comprising 13% of the isolates in the dataset. The remaining isolates (*n* = 6377 isolates) were distributed among 648 multi-isolate clusters (i.e., ≥ two isolates). The number of isolates per cluster ranged from 2 to 398, with clusters of 10 or more isolates (*n* = 121 clusters) comprising over 64% of the isolates (*n* = 5207/8089 isolates) in the dataset.

### 2.3. Cattle Were the Dominant Source of Attributable Human Campylobacteriosis Cases

The source attribution estimates were obtained using two different reference CGF40 databases, a SWA-only database and a pan-Canadian database (i.e., the Canadian *Campylobacter* CGF40 database [C3GFdb]). The source attribution estimates computed using the subtyping data from SWA only were found to be: ≈80% for cattle, with a 95% confidence interval (CI) [79.9, 80.1]; ≈8.7% for chicken, with a 95% CI [8.4, 9.0]; and ≈11.2% for water, again with a 95% CI [11.1, 11.3]. The attribution estimates computed using the subtyping data within the C3GFdb were found to be: ≈68% for cattle, with a 95% CI [67.9, 68.1]; ≈21.1% for chicken, with a 95% CI [21.0, 21.2]; and ≈9.2% for water, again with a 95% CI [8.1, 10.3]. Under the null hypothesis, the proportion of human clinical cases attributable to chicken would be expected to be significantly higher than that attributable to cattle. Using attribution estimates based on the SWA reference database, for cattle (≈80%, 95% CI [79.9, 80.1]) versus chicken (≈8.7%, 95% CI [8.4, 9.0]), the null hypothesis was rejected (*p* < 0.001). Using estimates based on the pan-Canadian C3GFdb, for cattle (≈68%, 95% CI [67.9, 68.1] versus chicken (≈21.1%, 95% CI [21.0, 21.2]), the null hypothesis was also rejected (*p* < 0.001).

### 2.4. A High Proportion of Campylobacter jejuni Isolates Were in CGF40 Clusters Comprising Isolates from Multiple Sources

**Figure 2:**
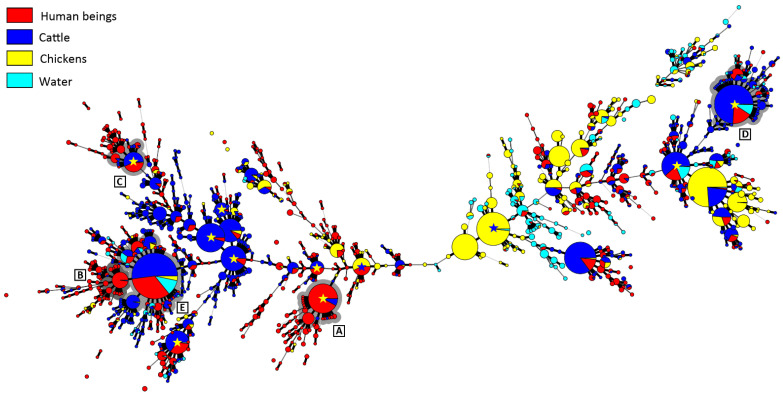
Minimum spanning tree of *Campylobacter jejuni* comparative genomic fingerprinting (CGF40) subtypes (*n* ≥ two isolates) recovered from people, chickens, cattle, and water in southwestern Alberta (SWA) from 2004–2018. Red fill denotes isolates from people, yellow fill denotes isolates from chickens, dark blue fill denotes isolates from cattle, and light blue fill denotes isolates from water. The thickness of lines connecting subtypes represents mismatched loci (i.e., one to three loci), and subtypes with no line represent ≥ four mismatched loci between respective subtypes. Yellow stars indicate prominent subtypes in which isolates within the subtype have been isolated from chickens elsewhere in Canada (i.e., queried against fingerprinted *C. jejuni* isolates within the Canadian *Campylobacter* CGF40 database). Blue stars indicate prominent subtypes in which isolates within the subtype have been isolated from cattle elsewhere in Canada. Grey highlighted clusters illustrate clusters containing large numbers of clinical *C. jejuni* isolates (95% level of CGF40 resolution). Eight thousand and eighty-nine *C. jejuni* isolates from SWA were fingerprinted in the study, but the 1773 *C. jejuni* isolates that formed singletons are not included in the figure.

**Figure 3:**
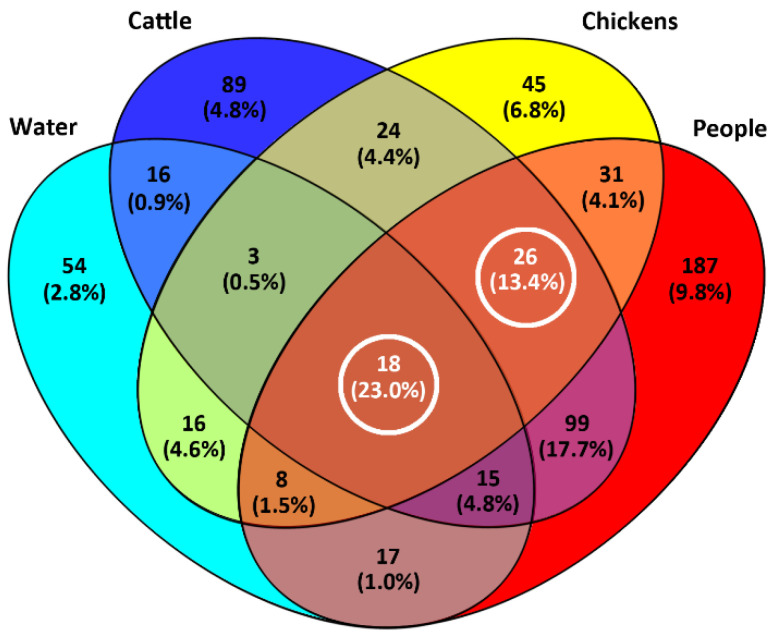
Diversity of all *Campylobacter jejuni* comparative genomic fingerprinting (CGF40) clusters (95% level of resolution) recovered from people, cattle, chickens, and water in southwestern Alberta (SWA) from 2004 to 2018 queried against data within the Canadian *Campylobacter* CGF40 database. Numbers depict the total number of CGF40 clusters, and values in parentheses represent the percentage of isolates in SWA that are unique and shared among sources.

Although in excess of 84% of the CGF40 clusters (*n* = 1439/1712 clusters) were unique to a single source that included singleton clusters (*n* = 1064 clusters), these comprised only 34% (*n* = 2761/8089 isolates) of the *C. jejuni* isolates included in the study. Thus, the majority of isolates (66%; *n* = 5328/8089 isolates) were contained in multi-isolate CGF40 clusters (*n* = 273 clusters) that included isolates from multiple sources (Figure 2). More specifically, multi-source CGF40 clusters were composed of isolates from two sources (*n* = 203 clusters; *n* = 2294/8089 isolates), three sources (*n* = 52 clusters; *n* = 1415/8089 isolates), and four sources (*n* = 18 clusters; *n* = 1619/8089 isolates) (Figure 3).

### 2.5. Cattle Was the Dominant Source of Clinically Relevant CGF40 Clusters

**Figure 4:**
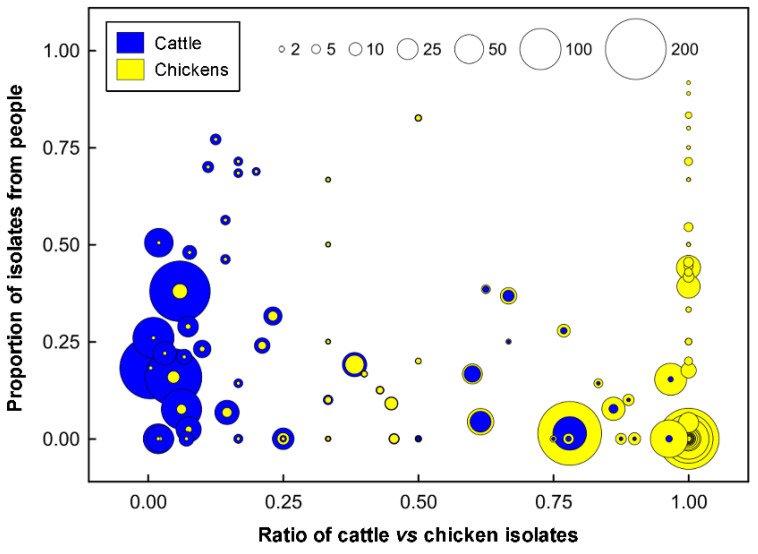
Degree of cattle and human source association among chicken *Campylobacter jejuni* comparative genomic fingerprinting (CGF40) clusters (95% level of resolution) observed in southwestern Alberta. The x-axis shows the degree of chicken association, expressed as the ratio of the number of isolates from cattle (blue) versus the number of isolates from chickens (yellow). The y-axis shows the level of human clinical association, where the number of clinical isolates is shown as a proportion of the total number of isolates in that CGF40 cluster. The size of each node is representative of the aggregate number of isolates from each source in a particular cluster, and a cluster size scale is included at the top of the graph.

**Figure 5:**
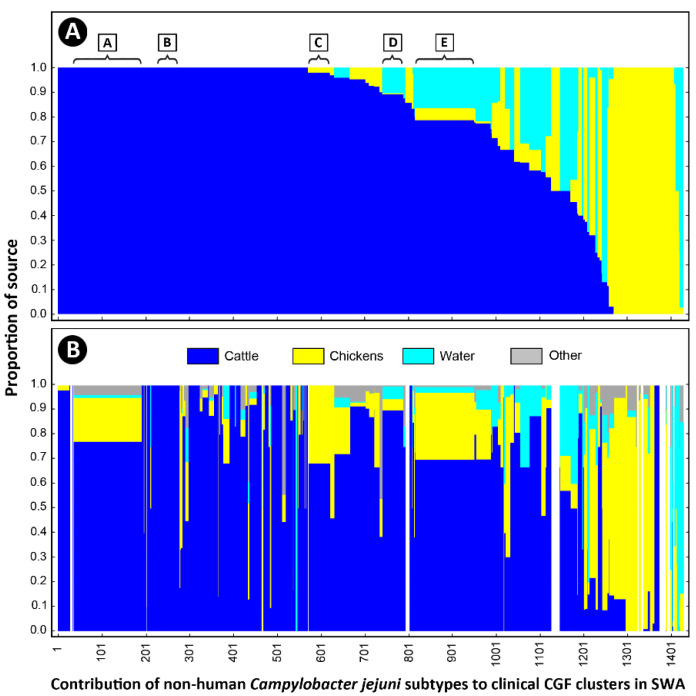
Source attribution for human campylobacteriosis cases observed in southwestern Alberta (SWA) from 2004 to 2018. Of the 812 comparative genomic fingerprinting (CGF40) clusters (95% level of resolution) observed among *Campylobacter jejuni* isolates recovered for people in SWA, 214 clusters representing 56.9% of human isolates matched with clusters containing isolates from non-human sources, including water, cattle, and chickens, and are included in the figure. (**A**) The relative contribution of non-human sources in SWA to each of the 214 clinical subtype clusters of *C. jejuni* in SWA. (**B**) The relative contribution of non-human sources in Canada (i.e., within the Canadian *Campylobacter* CGF40 database) to each of the 214 clinical subtype clusters of *C. jejuni* in SWA. CGF40 clusters indicated with parentheses and marked A–E denote clusters containing large numbers of clinical *C. jejuni* isolates observed in SWA (A and B containing isolates exclusively from people and cattle, and C–E containing isolates that are from people, cattle, and other sources). Prominent CGF40 clusters A–E are also shown in Figure 2.

An examination of the isolates recovered from individuals with campylobacteriosis (*n* = 2548 isolates) showed that they were broadly distributed among 813 CGF40 clusters (i.e., “Clinically Relevant Clusters” [CRCs]). These encompassed 599 CRCs unique to people (*n* = 1098 isolates), including 412 singleton CRCs that did not have matching subtypes among non-human sources. Two hundred and fourteen “source-associated” CRCs, which contained nearly 57% of the human clinical *C. jejuni* isolates examined in the study (*n* = 1450/2548 isolates), included isolates from both people and non-human sources (Figure 3, Supplemental Table S2). Notably, nearly 88% of source-associated human isolates (*n* = 1270/1450 isolates) belonged to CRCs (*n* = 158 clusters) that included cattle isolates. In contrast, ≈51% (*n* = 741/1450 isolates) were in CRCs that included chicken isolates (*n* = 83 isolates) and ≈36% (*n* = 522/1450 isolates) were in CRCs that included water isolates (*n* = 58 isolates). Moreover, 68% of source-associated human isolates (*n* = 991/1450 isolates) were distributed in 120 CRCs in which cattle was the predominant non-human source (i.e., clusters in which >75% of the non-human isolates were from cattle), whereas those in chicken-predominant and water-predominant clusters were ≈11% (*n* = 163/1450 isolates distributed in 37 CRCs) and ≈3% (*n* = 47/1450 isolates distributed in 21 CRCs), respectively (Figure 4). Similarly, although CRCs (*n* = 44 clusters) that included isolates from both cattle and chickens represented over 40% of source-associated human isolates (*n* = 582/1450 isolates), the majority of these (*n* = 373/582 isolates) were in CRCs (*n* = 16 clusters) in which cattle was the predominant non-human source. This included five prominent CRCs with the largest number of human clinical isolates in this study (Figure 2 and Figure 5A). Source associations were also computed using the C3GFdb, and were found to be ≈69%, ≈21%, and ≈7% for cattle, chicken, and water, respectively (Figure 5B).

### 2.6. Additional Evidence Supports Beef Cattle as an Important Reservoir of Campylobacter jejuni Subtypes Infecting Chickens

**Figure 6:**
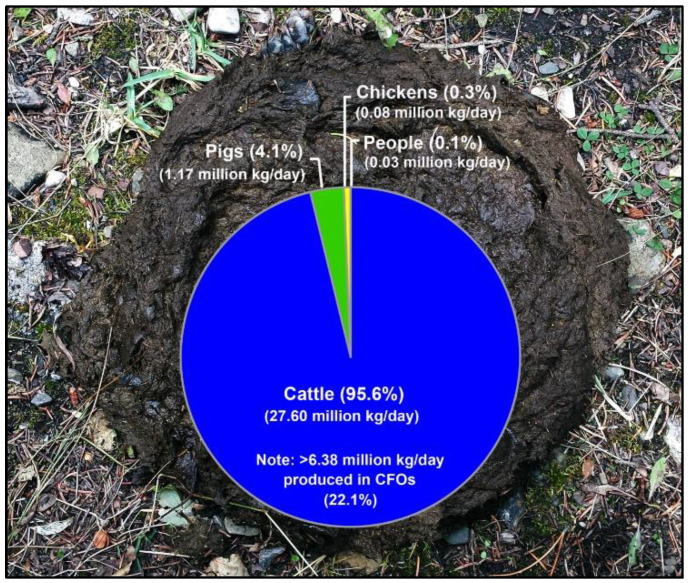
Cow pie chart showing relative manure production (dry weights) by primary livestock species and people in southwestern Alberta. Manure production assumes that feeder cattle are 295 kg, other cattle are 590 kg adults, grower hogs are 52 kg, adult hogs are 180 kg, layer chickens are 1.6 kg, broiler chickens are 0.5 kg, and people are 68 kg on average. Sources used were the 2011 Census of Agriculture for Alberta [17], Statistics Canada [20], USDA Natural Resources Conservation Service Agricultural Waste Management Handbook [21], and the 2011 Municipal Affairs Population List for Alberta [18].

**Figure 7:**
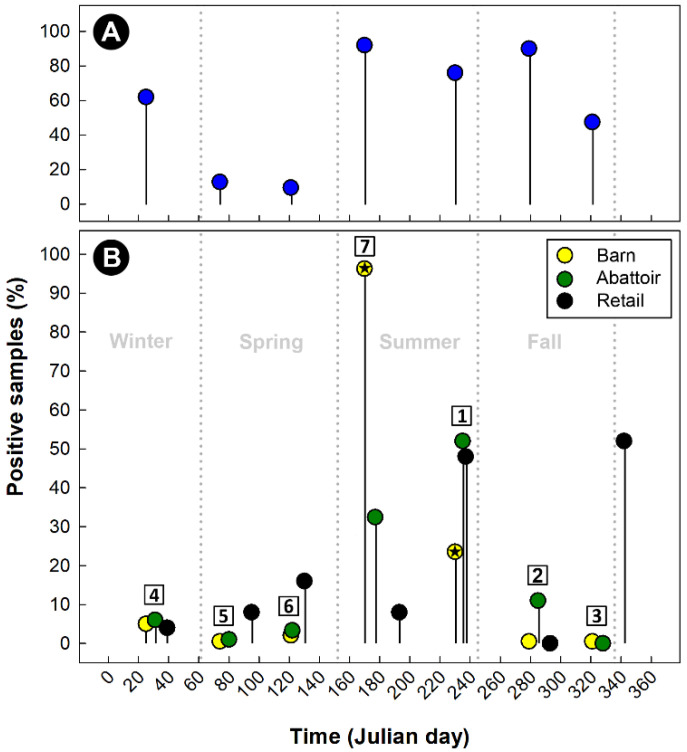
Seasonal prevalence of samples positive for *Campylobacter jejuni* (%). (**A**) Beef cattle feces. (**B**) Poultry samples in a broiler barn, the abattoir at which the birds were processed, and at retail [22]. The broiler barn and beef cattle confined feeding operation sampled were located within 1.0 km from each other (Figure 8). Numbers within squares represent the broiler production cycle sampled within the same barn over a 1-year period (*n* = 7 cycles). Markers with a star indicate outbreaks of *C. jejuni* within the broiler barn (i.e., >20% of birds infected with the bacterium). Julian day 1 is January 1st. Genotypic information of the *C. jejuni* isolates recovered from poultry, beef cattle, and human beings during the study period is presented in Figure 8 and Supplemental Table S3.

**Figure 8:**
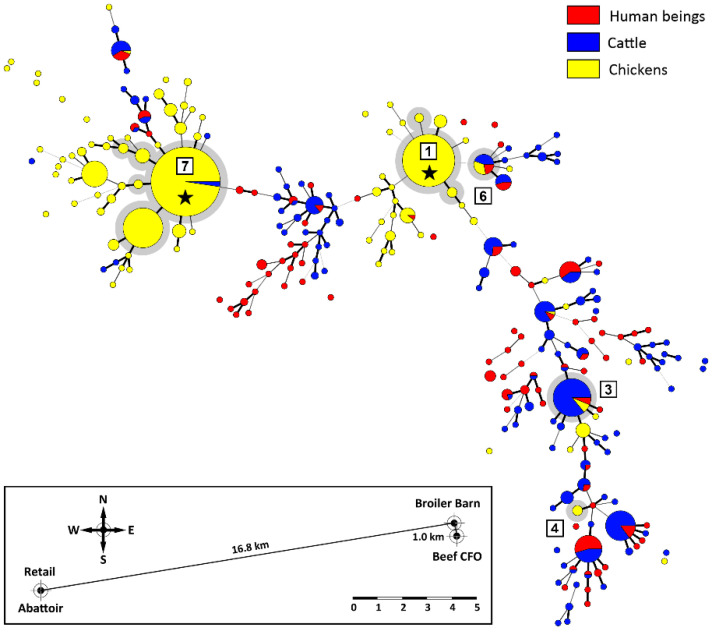
*Campylobacter jejuni* comparative genomic fingerprint (CGF40) subtypes recovered from a broiler barn, the abattoir at which the birds were processed, and retail poultry (yellow) over a 1-year period in southwestern Alberta (seven cycles). In addition, *C. jejuni* CGF40 subtypes recovered from beef cattle (blue) from an adjacent confined feeding operation (CFO), and from people living in SWA during the same time period (red) are presented. CGF40 subtype clusters (95% level of resolution) with grey shading and a number within a square reference the production cycle, and clusters denoted with a star indicate outbreaks of *C. jejuni* within the broiler barn. The minimum spanning tree was generated in Bionumerics (version 6.6, Applied Maths). The size of the circles is proportional to the number of *C. jejuni* isolates within the subtype. The thickness of lines connecting subtypes represent mismatched loci (i.e., one to three loci), and subtypes with no line represent ≥ four mismatched loci between respective subtypes. The bottom insert shows the relative position of beef cattle CFO, the broiler barn, the abattoir at which birds were processed and sold at retail in SWA. The scale bar is in km. The prevalence of *C. jejuni* isolates associated with chickens and cattle is presented in Figure 7, and information on subtype clusters 3, 6, and 7, which contained isolates from both chickens and cattle is presented in Supplemental Table S3.

Cattle are the predominant producers of feces in SWA, responsible for 95.6% of the total fecal output in the region (Figure 6). In contrast, pigs (4.1%), chickens (0.3%), and people (0.1%) produce substantially less feces. Of the 789 beef cattle fecal pats that were sampled over a 1-year period, 400 (50.7%) were culture positive for *C. jejuni* (Figure 7A). The prevalence of isolation varied over the sample period, with recovery rates ranging from 9.6% to 92.0% in the spring and summer, respectively. The high prevalence of cattle pats positive for *C. jejuni* corresponded to the outbreak of infections in the broiler barn adjacent to the beef cattle CFO, and subsequently of the meat in the abattoir and the meat sold at retail (Figure 7B). In excess of 1000 *C. jejuni* isolates were subtyped by CGF40 (*n* = 608 isolates associated with chickens, *n* = 320 isolates from cattle feces, and *n* = 164 isolates from stools from people). A considerable diversity of *C. jejuni* isolates were recovered from beef cattle fecal pats (Figure 8). In several instances, the subtypes recovered from cattle were also recovered from chickens housed in the adjacent broiler barn. In this regard, three prominent *C. jejuni* subtypes (0238.007.002, 0853.008.001, and 0735.001.002) were recovered from the cattle and from the adjacent chicken barn in cycles 3, 6, and 7 (Figure 7 and Figure 8, Supplemental Table S3). The *C. jejuni* subtypes 0238.007.002, 0853.008.001, and 0735.001.002 correspond to the multi-locus sequence typing (MLST) sequence types (STs) 806, 933, and 459 (Clonal Complexes ST-21, ST-403, and ST-42, respectively). Notably, all three of these subtypes have been reported previously in people and cattle in Alberta and elsewhere in Canada, and two of these subtypes (0238.007.002 and 0853.008.001) were also recovered from people during the study period.

### 2.7. A Model for the Indirect Transmission of Bovine Campylobacter jejuni to People via Contamination of the Chicken Supply Chain

**Figure 9:**
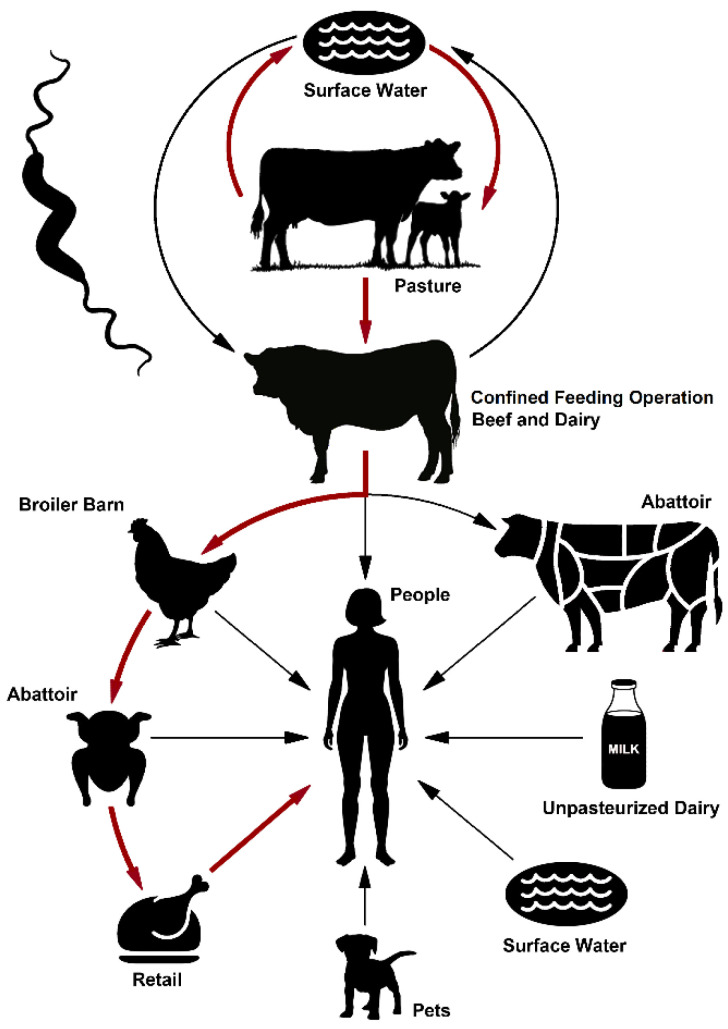
Primary (red arrows) and secondary (black arrows) transmission pathways of *Campylobacter jejuni* in southwestern Alberta.

The findings of the current study using SWA as a model agro-ecosystem, in conjunction with previously published evidence, support cattle as a primary reservoir of clinically relevant subtypes of *C. jeuni*, and a transmission pathway from cattle to chickens to human beings (Figure 9). In this regard, the accumulated evidence indicates that *C. jejuni* circulates in cattle on a pasture, and within cattle in CFOs. The *C. jejuni* cells excreted in cattle feces are transmitted to a broiler barn, within which a generalist clinically relevant subtype of the bacterium results in an outbreak within chickens housed in the barn. The infected chickens within an individual barn subsequently introduce *C. jejuni* into the chicken production continuum, and chicken contaminated with clinically relevant subtypes of the bacterium originating from cattle reservoirs infect people during the preparation and consumption of poultry.

### 2.8. Global Production of Chickens and Cattle Occurs in the Same Geographical Space

Chicken production for eggs and meat, and cattle production for meat and dairy occurs in the majority of countries globally (Supplemental Figure S4A–C), although the husbandry strategies that are utilized vary by country. In this regard, the concentration of dairy and beef cattle production in CFOs is a common practice in North America and Europe, although the relative densities of cattle versus chicken production vary by country (Figure S5, Supplemental Table S4). Some countries rank highly in cattle production but low in chicken production (e.g., some African countries), but many countries rank highly for both livestock. In North America, the United States ranks 3rd in cattle densities (94.8 M animals) and 3rd in densities of chickens (1972.3 M animals), Mexico ranks 8th in cattle densities (35.2 M animals) and 8th in densities of chickens (580.3 M animals), and Canada ranks 31st in cattle densities (11.5 M animals) and 29th in densities of chickens (171.4 M animals). In most countries, cattle and chicken production is spatially concentrated in certain locations. In Canada, for example, cattle and chicken farms are concentrated in specific regions of the country, particularly in southern British Columbia, Ontario, and Quebec (Supplemental Figure S1B), although both livestock species are farmed in other regions of Canada, such as in SWA. It is noteworthy that dairy, broiler meat, and egg production are supply managed in Canada (i.e., the production is managed by marketing boards under quotas provided to producers), whereas beef cattle production is not. This influences the numbers and sizes of farms producing dairy, broiler meat, and eggs in Canada.

## 3. Discussion

A multitude of studies have shown that the consumption of inappropriately prepared chicken is the primary mechanism of the transmission of *C. jejuni* to human beings [23,24]. The southwestern region of Alberta possesses high rates of campylobacteriosis, ranging from 43 to 94 infections/100 K, although the application of enhanced isolation methods has shown the disease to be substantially higher in the region (i.e., >115 infections/100 K) [1]. Despite the high rates of campylobacteriosis in SWA relative to other regions of the province, there is no evidence to indicate that chicken is contaminated with *C. jejuni* to a higher degree than elsewhere [22], or that the chicken consumption habits differ across the province of Alberta.

A salient characteristic of SWA is the high density of cattle in the region, along with chicken broiler and layer production in CFOs. At any given time, there are up to 1.1 M head of cattle in the region, with approximately 50% being housed in CFOs situated in the region of SWA where the majority of people live. Notably, there are substantially more cattle in SWA than people (ratio of ≈7:1). Several lines of evidence, including multiple source attribution studies, have consistently shown that bovine species such as cattle and sheep rank second to chicken in terms of the relative contribution to human campylobacteriosis cases. We have previously shown that the *C. jejuni* subtypes associated with cattle, including strains resistant to key antibiotics, are often observed among human cases of campylobacteriosis in SWA [1,12].

To investigate the role of cattle as a source of strains infecting people in SWA, we subtyped >8000 *C. jejuni* isolates recovered from people, cattle, chickens, and water in order to perform a microbial subtyping-based source attribution [25]. The isolates were comparatively analyzed using CGF40, a high throughput and high-resolution fingerprinting method that targets 40 accessory loci in the *C. jejuni* chromosome [26]. Notably, the CGF40 method has been shown to provide a greater discriminatory power than conventional multi-locus sequence typing [26,27], an essential feature for frequency matched attribution models, which require a subtyping method that balances sufficient discrimination and the capability to observe subtypes among both human and non-human sources [28]. In addition, we have established and curate a national database that contains data on >28,000 *C. jejuni* isolates recovered from various sources from across Canada. This information on *C. jejuni* subtypes circulating in a pan-Canadian context can greatly facilitate the interpretation of the study’s findings.

A considerable genotypic diversity was observed among the *C. jejuni* isolates examined in the current study, including 813 CGF40 clusters that included human clinical isolates (i.e., CRCs). Although only a minority of clusters (≈16%) included isolates from multiple sources, these comprised nearly two-thirds of the total isolates, including ≈57% of the isolates from people. To further examine the relative contribution of cattle, chickens, and water towards human cases of campylobacteriosis in SWA, the attribution estimates were computed using a basic Dutch source attribution model [29] using two different reference databases (i.e., SWA-only versus pan-Canadian C3GFdb). The number of cases attributable to cattle obtained using each database were shown to be significantly higher than the numbers obtained for chicken, including over nine-fold higher based on the SWA-only data, and over three-fold higher when using the pan-Canadian data. This discrepancy between both estimates can be explained by the much higher prevalence of cattle isolates in multiple prominent CRCs in the SWA surveillance data. Nonetheless, both estimates showed a disproportionate contribution of campylobacteriosis cases attributable to cattle with respect to chicken, which contrasts with the much lower estimates observed in other studies [8,9,11,30,31].

The role of cattle in the epidemiology of campylobacteriosis remains enigmatic and limited information currently exists on the routes of the transmission of *C. jejuni* from cattle to people. Previous research has shown that *C. jejuni* is commonly shed in beef and dairy cattle feces in SWA [32,33,34,35], and that cattle housed in CFOs shed a diversity of *C. jejuni* strains, including multiple strains from individual animals [14,15,36]. However, a longitudinal examination of the beef production continuum indicated that while *C. jejuni* was transmitted to dressed carcasses, the bacterium was not detected in the meat generated from the carcasses [14]. Moreover, viable *C. jejuni* is not commonly isolated from beef (intact and ground beef) sold at retail [8,37,38,39,40,41], suggesting that the foodborne risk of an infection via the consumption of beef or beef products is low. Although cattle have been linked to waterborne outbreaks of campylobacteriosis [42], a comparative examination of *C. jejuni* recovered from water and people over a 1-year period showed that river water was not a significant source of the subtypes of the bacterium infecting people in SWA [13]. Cattle have also been implicated through the ingestion of unpasteurized milk contaminated with the bacterium [43,44,45]. Occupational contact with cattle (e.g., in CFOs and abattoirs) has been identified as a potential risk factor for campylobacteriosis in SWA [46]. However, the cattle-associated pathways of transmission do not necessarily rank highly in terms of typical exposures [47], greatly complicating the identification of the transmission routes that disproportionately contribute to the burden of illness. Thus, evidence indicates that although the *C. jejuni* subtypes associated with cattle are prevalent in people, the transmission is not via the direct foodborne or waterborne routes of exposure, which led us to investigate the hypothesis that cattle may serve as a primary reservoir for strains infecting people, and that infection is likely to involve alternate routes of transmission.

The systemic impact of cattle on the prevalence of the *C. jejuni* strains circulating in SWA can be evidenced from our molecular surveillance data. Nearly 88% of source-associated human isolates in the study were within CRCs that contained cattle isolates, with more than two-thirds belonging to clusters in which cattle isolates were the predominant non-human source. Importantly, the potential for the transmission of *C. jejuni* emanating from cattle to chicken to people is also evident in these data, with ≈64% of the human isolates found within CRCs that contained isolates from chicken and cattle within cattle-predominant CRCs. The molecular epidemiology of *C. jejuni* in broiler chickens has previously been examined in SWA [22]. In this regard, a detailed longitudinal examination of the transmission of *C. jejuni* throughout the broiler production continuum revealed that an infection in broiler chickens by *C. jejuni* was a relatively rare event, with barn outbreaks incited by a predominant strain. Moreover, the diversity of the *C. jejuni* subtypes increased substantially at the abattoir, including subtypes not previously observed in the barns that were surveyed [22]. Significantly, many of the *C. jejuni* strains observed in the broiler production continuum in SWA were primarily associated with cattle [22]. A high proportion of cattle-dominant CRCs in the pan-Canadian molecular surveillance data of the chicken production continuum has also been observed in the most recent national baseline study of broiler chickens undertaken by the Canadian Food Inspection Agency (unpublished data).

In the current study, we examined the temporal prevalence of *C. jejuni* associated with broiler chickens and beef cattle housed in a CFO adjacent to the broiler barn (within ≈1 km). *Campylobacter jejuni* was commonly recovered from the fecal pats of cattle housed in the CFO, and the highest prevalence of fecal pats positive for *C. jejuni* occurred during the summer and fall months, which corresponded to outbreaks in the adjacent broiler barn, and with contamination of the meat within the abattoir. In addition, we observed that the same *C. jejuni* subtype associated with beef cattle and broilers was present in the chicken broiler barn adjacent to the cattle CFO. The three prominent *C. jejuni* subtypes recovered from cattle and the adjacent chicken barn are commonly associated with cattle and people in Alberta and elsewhere in Canada. Moreover, these CGF40 subtypes (0238.007.002, 0735.001.002, and 0853.008.001) correspond to STs within clonal complexes, ST-21, ST-42, and ST-403, respectively, which in addition to being associated with human clinical cases, have been associated with multiple non-human sources, including cattle and chickens within the pubMLST database [48]. The transmission of *C. jejuni* from the bovine reservoir to chickens in broiler barns may occur through a number of potential mechanisms. Flying and ground-dwelling arthropods are able to transmit *C. jejuni* [49,50], and arthropod activity corresponded with primary occurrences of outbreaks in broiler barns [24]. Furthermore, biosecurity measures precluding flying insects have been effective in managing *C. jejuni* in chickens [51,52,53]. The *C. jejuni* infections that we observed in SWA occurred primarily during summer and autumn, consistent with the findings of other studies conducted in temperate climates [54,55]. In SWA, *C. jejuni* infiltrates broiler barns in sufficient numbers to incite outbreaks only infrequently, with outbreaks occurring late in the production cycle [22]. Despite a low frequency of broiler flock outbreaks, the large number of barns supplying centralized abattoirs resulted in the contamination of birds that had remained free of *C. jejuni* up to that point, facilitated by a passive transmission during transport [56]. Within an abattoir, the risk of cross-contamination is increased due to the processing of a large number of broilers, including from different farms for which a small proportion of flocks are heavily contaminated with *C. jejuni*. It is currently unknown to what degree different subtypes persist within the abattoir environment, or the significance of the subtype’s persistence in the epidemiology of campylobacteriosis, but recent evidence suggests that survival differs amongst *C. jejuni* strains [57], which may result in the accumulation of diverse subtypes that include high-risk cattle-borne subtypes. As a result, diverse *C. jejuni* subtypes that contaminate chickens within the abattoir are conveyed to and persist on retail products, subsequently infecting people that consume chicken that is improperly prepared.

Our findings indicated that the circulation of *C. jejuni* subtypes capable of transmission among cattle, chickens, water, and people in SWA are consistent with the “generalist” genetic lineages previously described [58,59]. The findings of the current study, in conjunction with previously published evidence, support cattle as a primary amplifying reservoir of clinically relevant subtypes. More specifically, in addition to a possible transmission through an occupational exposure, the data are consistent with multiple indirect transmission pathways through the widespread dissemination of clinically relevant subtypes of bovine origin throughout the agro-ecosystem via a contamination of surface waters and the chicken production continuum. Cattle on a pasture (i.e., cow–calf operations) are permitted to access surface waters, with both dairy and beef cattle requiring large quantities of water; a lactating beef cow on a pasture consumes 55 L/day on average [60]. Thus, cattle frequently congregate in riparian zones, depositing manure in or in proximity to surface waters, and a percentage of calves maintained on a pasture are colonized by *C. jejuni* via the consumption of contaminated water and/or feces. Upon a transfer to a CFO, the cattle colonized by *C. jejuni* on a pasture rapidly transmit the bacterium horizontally, resulting in the majority of animals becoming persistently colonized by multiple *C. jejuni* subtypes, including high-risk subtypes capable of colonizing multiple hosts (i.e., “generalist” subtypes) [58]. Diverse *C. jejuni* subtypes are chronically excreted in feces, where they have the potential to persist extra-intestinally for prolonged periods in beef manure [61], likely facilitating inter-animal transmission. Significant quantities of feces accumulate from cattle housed in CFOs, which are typically not removed from paddocks until the animals depart the CFO. It is recommended for manure to be composted before it is spread onto soil, although the distribution of raw manure onto soil with restrictions is permitted in Alberta [62] and other jurisdictions globally.

Critically, both the previous and current data are consistent with the introduction of *C. jejuni* subtypes into the broiler production chain, which subsequently infect the human population through the consumption of contaminated chicken products (Figure 9). Although the potential role that cattle-borne *C. jejuni* may play in the infection of chickens has been raised previously by others [63,64,65,66,67,68], to our knowledge, the current study is the first to provide molecular evidence suggesting a directionality of the transmission from cattle to chickens to people given the significant predominance of these subtypes in cattle in the surveillance data. At present, conclusive direct evidence of the transmission between cattle and chickens, and of the mechanisms involved, is limited. Our evidence implicating cattle as the primary amplifying reservoir of *C. jejuni* subtypes that infect people via a contamination of the chicken production chain obtained from SWA, Canada as a model location will undoubtedly raise the question of whether this transmission pathway is unique to this location. It is noteworthy that two recent studies suggest a potentially higher contribution of cattle towards human infections in data from France as compared to the United Kingdom [69,70]. Moreover, other locations in Canada, and the majority of countries globally, contain large populations of both chickens and cattle (beef and dairy), with production often being concentrated spatially in certain locations. The validation of the transmission pathway proposed in this study will be necessary in other locations. In this regard, the formulation of mechanistic hypotheses that are tested using empirical experimentation, as well as the application of molecular epidemiological strategies, including whole genome sequence data, is needed. We hope that the evidence we present herein in support of a transmission pathway for *C. jejuni* from cattle to chickens to people will stimulate such an examination in other locations. Significantly, if cattle are confirmed to be a primary and widespread amplifying reservoir of *C. jejuni* infecting broilers and subsequently people, significant efforts should be placed on the elucidation of the primary transmission mechanisms of *C. jejuni* from cattle to chickens, which will facilitate the design, evaluation, and implementation of rationale-based strategies to disrupt the transmission pathway, and thereby reduce the burden of campylobacteriosis on people.

## 4. Materials and Methods

### 4.1. Campylobacteriosis Rates, and Livestock and Manure Production

The data on the campylobacteriosis rates, livestock densities, and livestock manure production by cattle, chickens, pigs, and people in Alberta were collated from a variety of sources [1,17,18,19,20,21,71,72]. The information on the global production of cattle meat, cattle dairy, and chicken production was obtained from the Food and Agriculture Organization of the United Nations [73].

### 4.2. Isolation of Campylobacter jejuni

All the *C. jejuni* isolates from people were recovered from stools submitted to the Chinook Regional Hospital (CRH) for a microbiological screening [1,74]; the stools were submitted at the direction of physicians for people who exhibited signs of enteric infection. At the CRH, a single method using Campy CVA Agar (Becton Dickinson, Oakville, ON, Canada) at 42 °C in an anoxic atmosphere was used. Additional *C. jejuni* isolates from human stool samples were recovered over a 1-year period using comprehensive isolation methods, including the use of a microaerobic atmosphere [1]. *Campylobacter jejuni* from chickens in SWA was isolated from retail meat, broiler chicken cloacae and feces, broiler barn environments, and chicken abattoirs using a combination of direct plating or enrichment isolation methods [22]. From cattle, isolates were obtained from the feces of beef and dairy cattle in CFOs by direct plating, and from carcasses and meat by a direct plating or enrichment, similarly to chickens [14,15,36]. In addition, isolates were obtained from the digesta harvested from the intestines of beef cattle. It is noteworthy that the information presented in Figure 7 and Figure 8 was obtained from broiler chickens in a production barn and from beef cattle housed in a CFO located 1.0 km from the broiler barn over a 1-year period (seven broiler production cycles). For each production cycle, cloacal samples were collected weekly from 75 arbitrarily selected live birds using mini swabs (Fisher Scientific Company, Ottawa, ON, Canada) (*n* = 2448 total cloacal samples). At weekly intervals (same collection times as the broiler barns), a subsample of feces (≈10 g) was collected from 25 arbitrarily selected fresh fecal pats of beef cattle housed in the adjacent CFO (*n* = 791 total fecal samples). The samples of the feces from individual pats were placed in tubes, maintained on ice for transport to the laboratory, and within 2 h of the collection, were streaked onto Karmali agar (Oxoid Canada, Nepean, ON, Canada) with selective supplement SR0167 (Oxoid Canada). In addition, *C. jejuni* in the air adjacent to the beef cattle CFO and boiler barn was determined using an inertia-type microbial air sampler (MAS 100; Millipore Canada Ltd., Etobicoke, ON, Canada) operated at 100 L of air/min for a 10 min period. The particles in the air were deposited directly onto Karmali agar with selective supplement SR0167 in a Petri dish placed in the sampler. *Campylobacter jejuni* was recovered from the surface waters of the Oldman River and its tributaries primarily by an enrichment [13]. With the exception of *C. jejuni* isolated at the CRH, the cultures were maintained in a microaerobic atmosphere (5% O_2_, 3% H_2_, 10% CO_2_, and 82% N_2_, or 5% O_2_, 30% H_2_, 10% CO_2_, and 55% N_2_) in anaerobic jars (Oxoid Canada) at 37 °C or 42 °C. Presumptive *C. jejuni* isolates were selected based on the colony morphology, cell shape and size, and motility, were streaked for purity, and the biomass was stored in Columbia broth containing 40% glycerol at −80 °C until identified.

### 4.3. Identification of Campylobacter jejuni

*Campylobacter jejuni* isolates were identified using molecular characteristics. In this regard, all the presumptive isolates were streaked for purity, and genomic DNA was extracted using an AutoGen 740 robot (AutoGen, Inc., Holliston, MA, USA) according to the manufacturer’s protocol for Gram-negative bacteria. To confirm the identity of the isolates, taxon-specific PCR targeting the *ipxA* gene [75], the *mapA* gene [76], and the *hipO* gene [77] were used. In instances where the identification was not definitive, the partial or near complete 16S rRNA gene was sequenced. The obtained sequence data were compared to the reference data within the National Center for Biotechnology [78] and the Ribosomal Database [79]. All the isolates were accessioned in the Intestinal Bacterial Collection at the Agriculture and Agri-Food Canada (AAFC) Lethbridge Research and Development Centre (LeRDC) and were stored at −80 °C and over liquid nitrogen or by lyophilization.

### 4.4. Subtyping of Campylobacter jejuni Isolates

All the *C. jejuni* isolates were fingerprinted using the CGF40 method as described previously [12,26]. Briefly, each isolate was subjected to eight multiplex PCR reactions that together assess the presence or absence of a set of 40 accessory gene targets found to have a variable carriage in the *C. jejuni* population and were used to generate a highly discriminatory binary fingerprint. The amplicons were resolved using a QIAxcel high throughput capillary electrophoresis system with a DNA Screening Kit (Qiagen Inc., Toronto, ON, Canada). The data were visualized using the BioCalculator v3.2 software (Qiagen Inc.) and they were converted to binary values based on the presence (1) or absence (0) of bands at each target amplicon, then compiled to create a CGF40 pattern. The resulting binary profiles were assigned to a CGF40 subtype derived from the clusters’ membership within the C3GFdb. A 95% similarity threshold was used to define the CGF40 clusters in this study. The subtype relationships were visualized using a minimum spanning tree produced with BioNumerics (version 6.6, Applied Maths, Austin, TX, USA).

### 4.5. Assignment of Sequence Type and Clonal Complex for CGF40 Subtypes

In order to derive the MLST designations corresponding to the CGF40 subtypes, ST and clonal complex exclusivity was computed based on in silico prediction of the MLST and CGF40 profiles using genome sequence data obtained from the National Center for Biotechnology Information’s Sequence Read Archive. In silico MLST and CGF40 predictions were generated using the scripts *mlst* (https://github.com/tseemann/mlst, accessed on 11 November 2022) and CGFPrediction (https://github.com/sfisher4/CGFPrediction, accessed on 11 November 2022), respectively. Among the genomes analyzed, only those with complete in silico MLST and CGF40 profiles were used in the subsequent analyses (*n* = 26,336 genomes). The multi-locus sequence typing exclusivity metrics were calculated by computing the proportion of genomes from a given CGF40 cluster that shared the most frequent ST or clonal complex designation; exclusivity metrics were only computed for the CGF40 clusters comprising a minimum of five genomes (*n* = 527 clusters; *n* = 23,131 genomes).

### 4.6. Source Attribution of Human Campylobacteriosis Cases

Source attribution was investigated using a basic ‘Dutch source attribution model’ [29]. Under the model, the number of human cases associated with subtype *X* and source *Y* was calculated by computing the product of the number of human isolates associated with subtype *X* in the study dataset and the fractional attribution of source *Y* in subtype *X*, which was based on the proportion of isolates from source *Y* and subtype *X* among the total number of non-human isolates of subtype *X* in a reference database comprising data on isolates from non-human sources. The total number of human cases attributed to source *X* was then computed by aggregating the totals obtained across all the subtypes observed in the dataset and the overall attribution estimate for source *X* was then calculated as the number of human cases attributable to source *X* as a proportion of the total number of attributable cases. The attribution estimates were computed using two different non-human reference databases, including a SWA-only database comprising the isolates collected as part of this study, and a historical pan-Canadian database (i.e., the C3GFdb). The attribution estimates were computed while applying a minimum cluster size threshold (*n* ≥ five) through which the cases associated with the subtypes in clusters below the threshold were excluded from the attribution estimates; these were included in the analysis as “unattributable” cases, a category that also included human isolates with subtypes that lacked matches in the non-human database.

### 4.7. Statistical Analysis of Source Attribution Estimates and Hypothesis Testing

A custom script was developed in the R language for statistical computing (version 4.1.1) [80] to derive source attribution estimates based on the Monte Carlo simulations. Random selections comprising 75% of the non-human isolates from each subtype were drawn 1000 times and summarized using the pooled means and standard deviations to calculate the attributable source fractions for each of the chicken, cattle, and water sources. This process was repeated on the sample of human isolates with 30 random draws of 500 isolates to estimate the overall attribution of the source for the human isolates included in this study. The analysis script and accompanying datasets are available online at https://github.com/hetmanb/CampySA_SWA, accessed on 11 November 2022). Under the null hypothesis, the proportion of human clinical cases attributable to chicken would be expected to be higher than those attributable to cattle; this hypothesis was evaluated by comparing the frequency distribution for cattle and chicken attribution estimates using a one-tailed *T*-test.

## 5. Conclusions

A large number of *C. jejuni* isolates from people, cattle, chickens, and water in a model agroecosystem were subtyped in order to quantify the contribution of various sources towards the burden of illness in the study location. The source attribution estimates indicated a disproportionate contribution of cattle towards cases of campylobacteriosis. Multiple lines of evidence implicated cattle as the primary amplifying reservoir of the *C. jejuni* subtypes infecting people via several indirect transmission mechanisms resulting from the systemic contamination of the study location by the bacterium shed in feces. Importantly, this included a significant exposure through the consumption of chicken contaminated with cattle-borne *C. jejuni.* As high densities of dairy and beef cattle occur in many regions of the world, including SWA, disrupting the cattle to chicken transmission pathway may effectively reduce morbidity in people.

## Data Availability

The data presented in this manuscript are available on request from the corresponding authors.

## Supplementary Materials

The following supporting information can be downloaded at: https://www.mdpi.com/article/10.3390/pathogens11111366/s1, Figure S1: Distribution and densities of primary livestock and people in counties of southwestern Alberta, and agricultural production areas, and densities of cattle and chicken farms in Canada; Figure S2: Campylobacteriosis rates incited by *Campylobacter jejuni* from 1987 to 2018 in Canada, Alberta, and southwestern Alberta, and regional rates of campylobacteriosis 2004–2006, and cartogram showing the relative human population size within each of the 17 health regions in Alberta; Figure S3: Temporal infections by *C. jejuni* in people living in southwestern Alberta over a 1-year study period (2008 to 2009); Figure S4: Average global production of cattle meat, average global production of cattle milk, and average global production of chicken meat by area; Figure S5: Global heat map of densities of cattle and chickens by area (i.e., cumulative rank); Table S1: Distribution of *Campylobacter jejuni* isolates from southwestern Alberta by year and source; Table S2: Distribution of *Campylobacter jejuni* isolates and comparative genomic fingerprinting subtype clusters by sampling origin in southwestern Alberta; Table S3: Comparative genomic fingerprinting subtypes of *Campylobacter jejuni* isolates recovered from broilers and beef cattle housed in a confined feeding operation adjacent to the broiler barn, and reference information within the Canadian *Campylobacter* comparative genomic fingerprinting database; Table S4: Global cattle and chicken inventories. References [1,17,18,71,72,73,81] are cited in the supplementary materials.
