# Supplementary material for: Molecular Epidemiological Evidence Implicates Cattle as a Primary Reservoir of Campylobacter jejuni Infecting People via Contaminated Chickens
Source: Pathogens. 2022 Nov 16;11(11):1366. doi: 10.3390/pathogens11111366 (PMC9698452; doi:10.3390/pathogens11111366)
Supplement: Supplementary file 1 [file pathogens-11-01366-s001.zip › pathogens-2007995-supplementary.pdf]

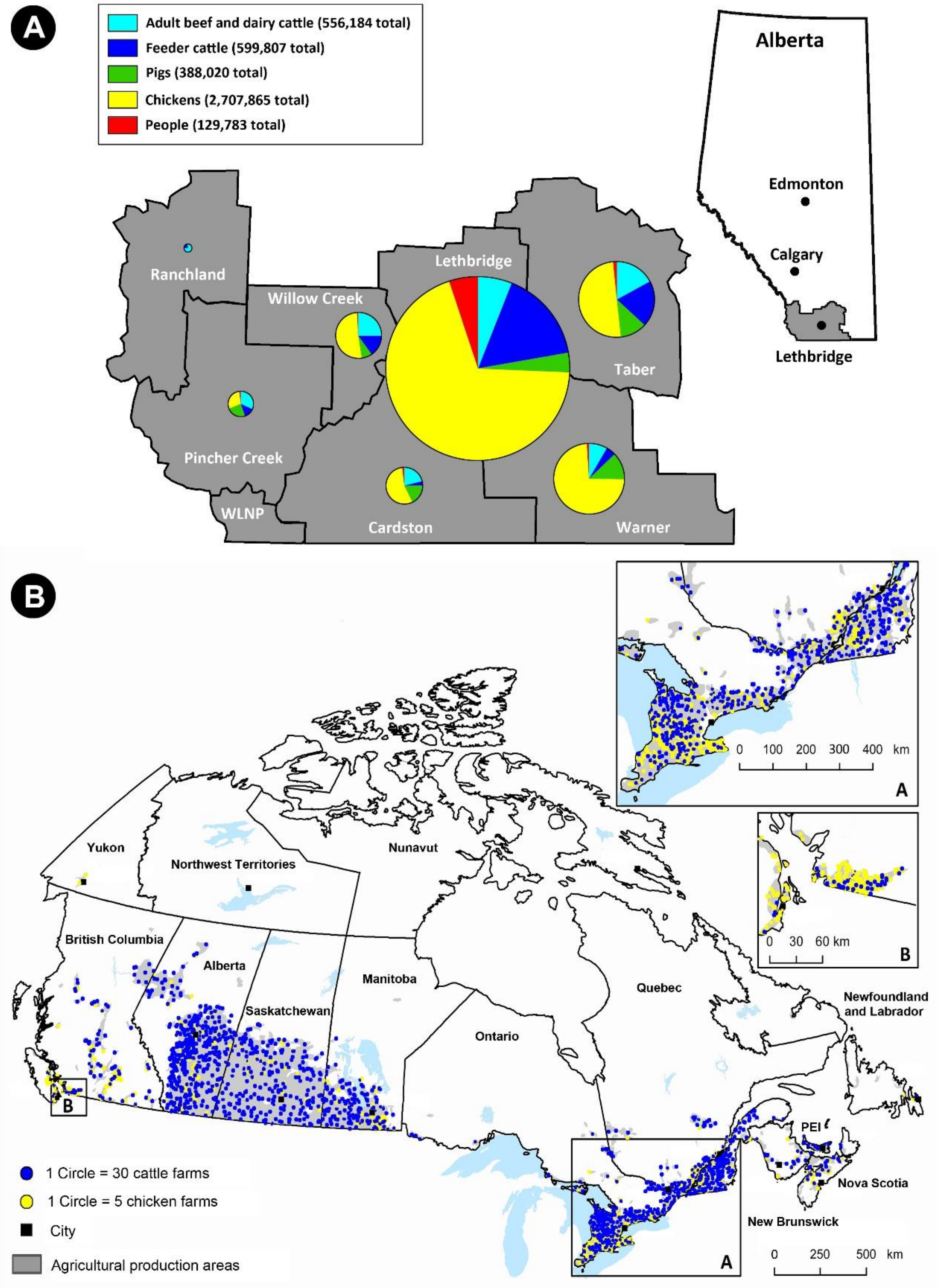


**Figure S1**. (**A**) Distribution and densities of primary livestock and people in counties of southwestern Alberta. The size of the pie chart represents the relative densities of animals. Livestock data was obtained from the 2011 Census of Agriculture for Alberta [1], and densities of people were obtained from the 2011 Municipal Affairs Population List [2]. (**B**) Agricultural production areas, and densities of cattle and chicken farms in Canada. Cattle farms include beef cattle and dairy confined feeding operations, as well as cow-calf farms. Data was obtained from Statistics Canada [3].

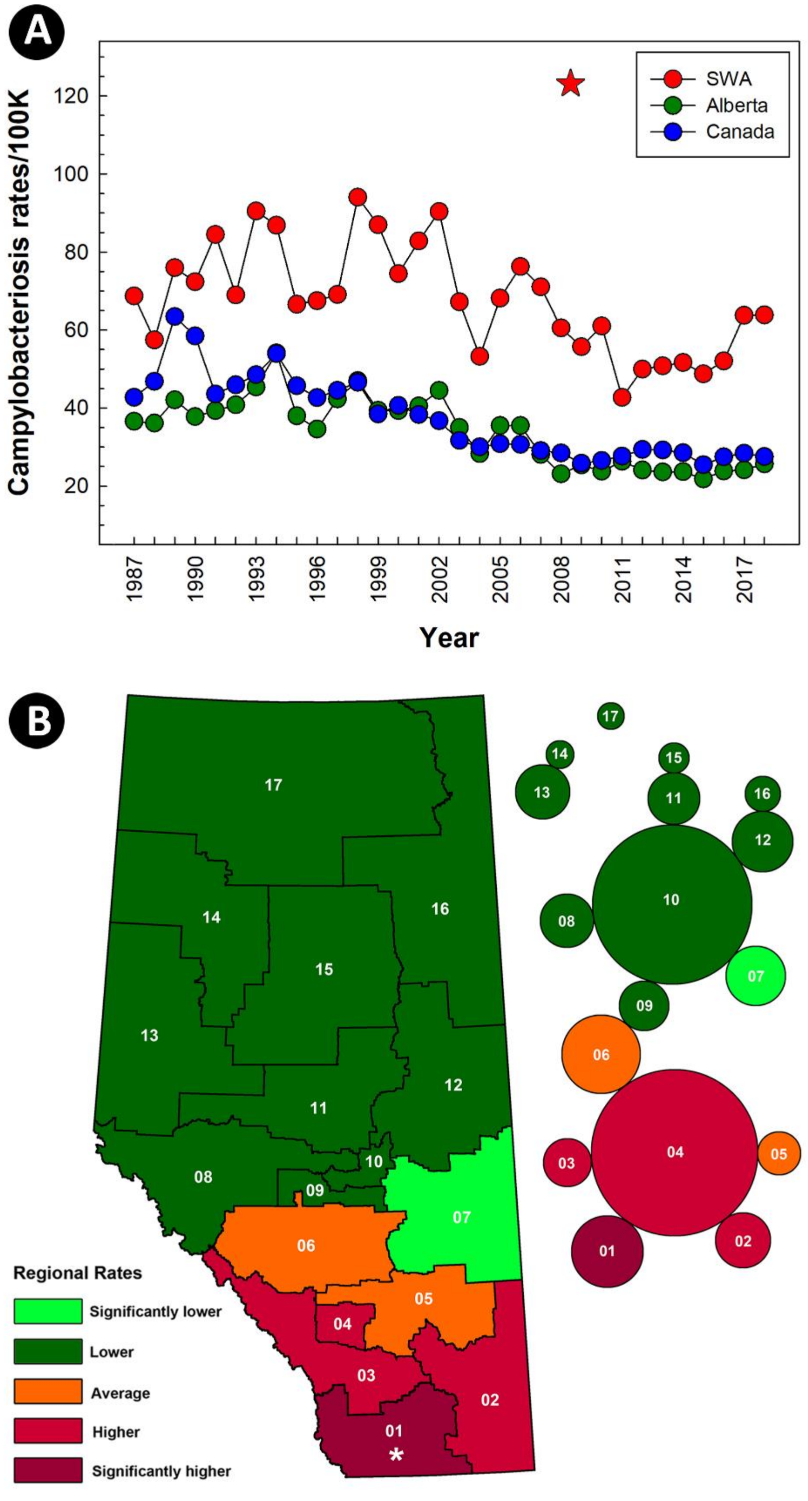


**Figure S2**. (**A**) Campylobacteriosis rates incited by *Campylobacter jejuni* from 1987 to 2018 in Canada [4], Alberta, and southwestern Alberta (SWA). The star indicates rate of campylobacteriosis determined by enhanced detection [5]. (**B**) Regional rates of campylobacteriosis 2004-2006 [6], and cartogram (right) showing the relative human population size within each of the 17 health regions in Alberta. The Asterisk indicates the SWA study location (i.e. health region 01).

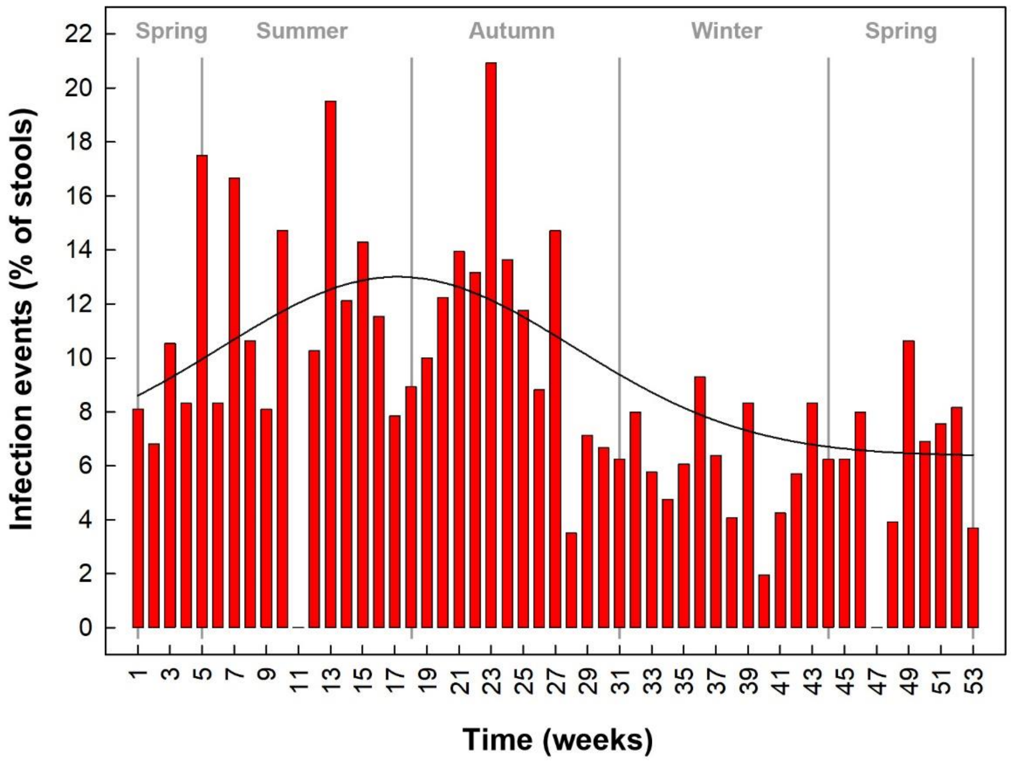


**Figure S3**. Temporal infections by *Campylobacter jejuni* in people living in southwestern Alberta over a 1-year study period (2008 to 2009). Stools were submitted to the Chinook Regional Hospital for microbiological screening by the physicians of people who exhibited signs of enteric infection. The curve was fitted to the prevalence data using the Dynamic Fit Wizard feature of SigmaPlot with the Gaussian, 4 Parameter option. The figure was generated from data published by Inglis et al. [5].

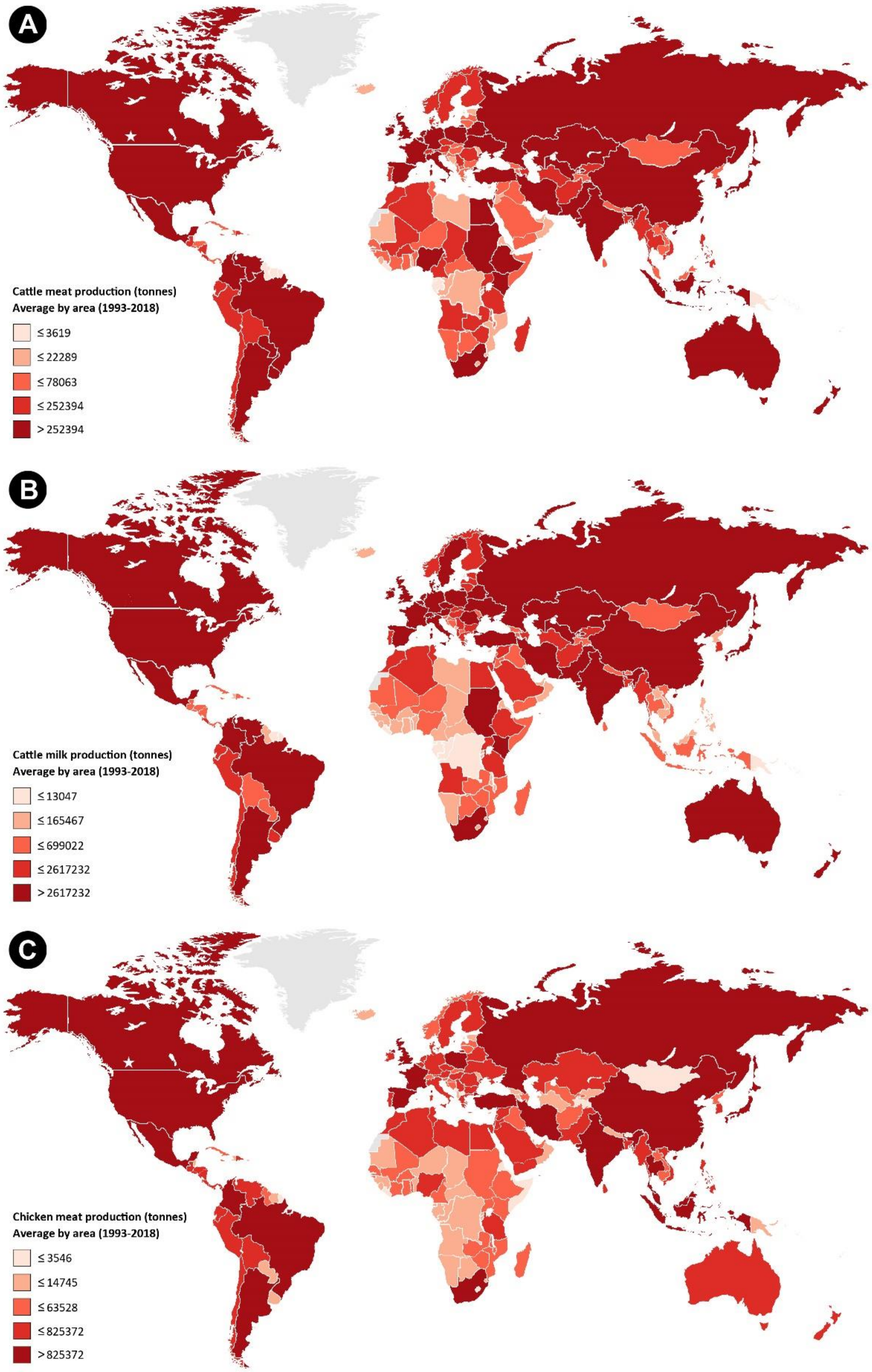


**Figure S4.** (**A**) Average global production of cattle meat by area. (**B**) Average global production of cattle milk by area. (**C**) Average global production of chicken meat by area. The star shows southwestern Alberta. The figure was generated from data obtained from the Food and Agriculture Organization of the United Nations [7].

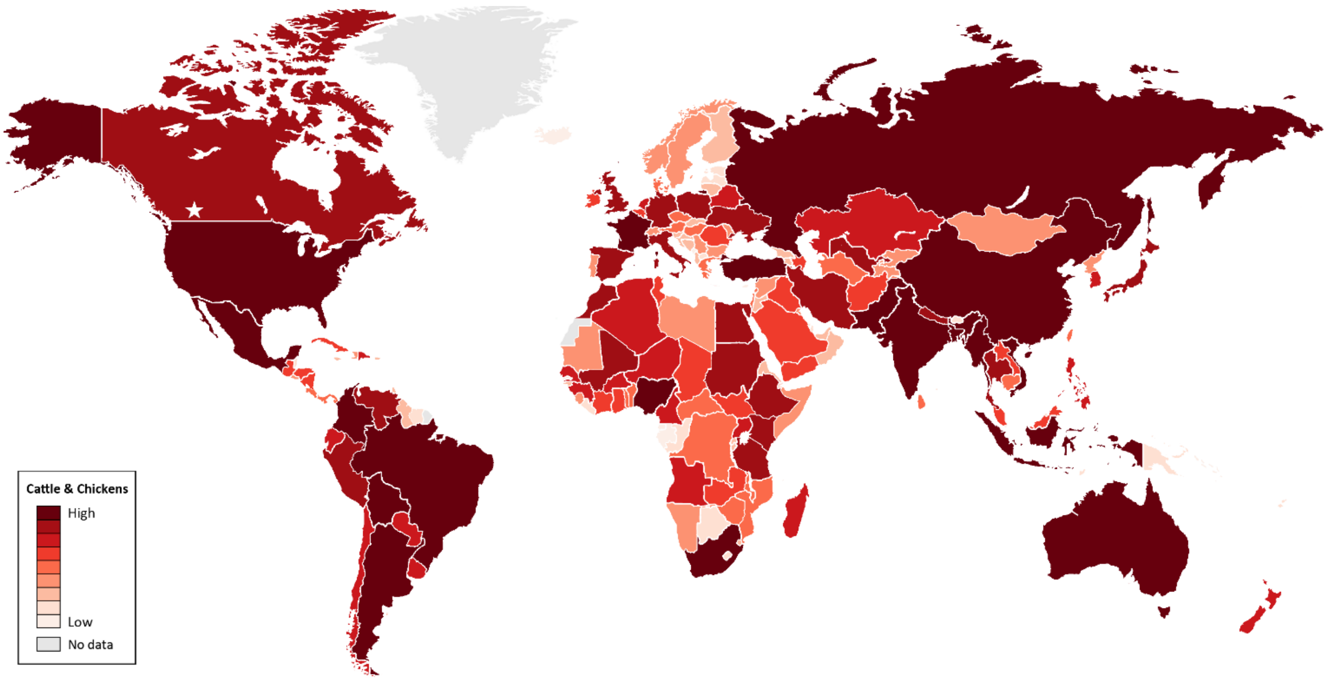


**Figure S5.** Global heat map of densities of cattle and chickens by area (i.e., cumulative rank). The star shows southwestern Alberta. The figure was generated from data obtained from the Food and Agriculture Organization of the United Nations [7]. Livestock densities and ranks are presented in Supplemental Table S4.

**Table S1.** Distribution of *Campylobacter jejuni* isolates from southwestern Alberta by year and source.

| Source | 2004 | 2005 | 2006 | 2007 | 2008 | 2009 | 2010 | 2011 | 2012 | 2013 | 2014 | 2015 | 2016 | 2017 | 2018 | Total |
|---|---|---|---|---|---|---|---|---|---|---|---|---|---|---|---|---|
| People | 80 | 103 | 109 | 389 | 851 | 290 | 88 | 65 | 77 | 75 | 73 | 70 | 79 | 106 | 93 | 2548 |
| Cattle | 0 | 598 | 0 | 0 | 470 | 1262 | 248 | 0 | 0 | 0 | 0 | 0 | 174 | 169 | 0 | 2921 |
| Chicken | 0 | 0 | 0 | 0 | 0 | 10 | 225 | 0 | 0 | 0 | 0 | 0 | 941 | 673 | 0 | 1849 |
| Water | 0 | 0 | 0 | 0 | 65 | 79 | 228 | 319 | 80 | 0 | 0 | 0 | 0 | 0 | 0 | 771 |
| Total | 80 | 701 | 109 | 389 | 1386 | 1641 | 789 | 384 | 157 | 75 | 73 | 70 | 1194 | 948 | 93 | 8089 |

**Table S2** Distribution of *Campylobacter jejuni* isolates and comparative genomic fingerprinting (CGF40) subtype clusters (95% level of CGF40 resolution) by sampling origin in southwestern Alberta.

| Category | People | Cattle | Chickens | Water | Total |
|---|---|---|---|---|---|
| No. isolates | 2,548 | 2,921 | 1,849 | 771 | 8,089 |
| No. CGF subtypes clusters | 813 | 553 | 416 | 291 | 1,712 |
| No. subtype clusters unique to origin (No. isolates) | 599 (1098) | 352 (597) | 290 (725) | 198 (341) | 1,439 (2,761) |
| % unique subtype clusters | 73.7 | 63.7 | 69.7 | 68.0 | 84.1 |
| No. subtypes common to other origins (No. isolates) | 214 (1,450) | 201 (2,324) | 126 (1,124) | 93 (430) | 272 (5,326)[1] |
| **Distribution of subtypes (isolates) common to[2]:** | | | | | |
| People | – | 158 (2,110) | 83 (713) | 58 (329) | |
| Cattle | 158 (1,270) | – | 71 (681) | 52 (266) | |
| Chickens | 83 (741) | 71 (1473) | – | 45 (276) | |
| Water | 58 (522) | 52 (910) | 45 (690) | – | |

[1]Different from the sum of the row because some subtypes were present in more than one origin.
[2]The number of subtype clusters and isolates within a column exceeds the number of common subtype clusters and isolates found in an origin because some subtype clusters were present in more than one other origin.

**Table S3** Comparative genomic fingerprinting (CGF40) subtypes of *Campylobacter jejuni* isolates recovered from broilers (within a broiler barn and abattoir at which the broilers were processed) and beef cattle housed in a confined feeding operation adjacent to the broiler barn, and reference information within the Canadian *Campylobacter* CGF40 database (C3GFdb). The information presented in the table corresponds to subtype information presented in Figure 8.

| | No of isolates | | | | | | | | Isolates of the same CGF subtype within the C3GFdb (%) | | | |
|---|---|---|---|---|---|---|---|---|---|---|---|---|
| **Cycle** | **Broiler Barn** | **Broiler Abattoir** | **Beef Cattle** | **People** | **Total** | **CGF subtype** | **MLST[1]** | **Cluster Size** | **Hu[2]** | **Ch[3]** | **Ca[4]** | **AB[5]** |
| 3 | 2 | 4 | 55 | 4 | 65 | 0238.007.002 | ST-806 | 73 | 19.2 | 8.2 | 63.0 | 64.4 |
| 6 | 5 | 1 | 8 | 4 | 18 | 0853.008.001 | ST-933 | 13 | 30.8 | 0.0 | 46.2 | 53.8 |
| 7 | 149[6] | 43 | 5 | 0 | 197 | 0735.001.002 | ST-459 | 76 | 5.3 | 0.0 | 85.5 | 93.4 |
| 7 | 17 | 29 | 0 | 0 | 46 | 0735.003.004 | Unknown | 5 | 0.0 | 0.0 | 100.0 | 100.0 |
| 7 | 7 | 6 | 0 | 0 | 13 | 0731.004.004 | Unknown | 16 | 6.3 | 0.0 | 50.0 | 100.0 |

[1]Multi-Locus Sequence Typing (ST; sequence type).
[2]Human beings.
[3]Chickens.
[4]Cattle.
[5]Alberta.
[6]A *C. jejuni* outbreak occurred during cycle 7 (see Figure 7).

**Table S4** Global cattle and chicken inventories (million [M] head) [7][1].

| Area | Cattle | | | Chicken | | | Cumulative |
|---|---|---|---|---|---|---|---|
| | Rank | No (M) | % | Rank | No (M) | % | Rank |
| China-Mainland | 4 | 63.39 | 4.20 | 1 | 5146.15 | 19.62 | 5 |
| Brazil | 1 | 214.66 | 14.21 | 4 | 1466.56 | 5.59 | 5 |
| United States of America | 3 | 94.80 | 6.27 | 3 | 1972.26 | 7.52 | 6 |
| India | 2 | 193.46 | 12.80 | 7 | 807.89 | 3.08 | 9 |
| Pakistan | 7 | 47.82 | 3.16 | 5 | 1321.00 | 5.04 | 12 |
| Mexico | 8 | 35.22 | 2.33 | 8 | 580.83 | 2.21 | 16 |
| Indonesia | 20 | 17.12 | 1.13 | 2 | 3725.21 | 14.20 | 22 |
| Russian Federation | 18 | 18.15 | 1.20 | 9 | 497.40 | 1.90 | 27 |
| Myanmar | 17 | 18.58 | 1.23 | 10 | 394.62 | 1.50 | 27 |
| Bangladesh | 14 | 24.19 | 1.60 | 14 | 286.90 | 1.09 | 28 |
| Turkey | 21 | 17.04 | 1.13 | 12 | 342.57 | 1.31 | 33 |
| Colombia | 12 | 27.24 | 1.80 | 22 | 187.50 | 0.71 | 34 |
| France | 19 | 18.15 | 1.20 | 17 | 237.86 | 0.91 | 36 |
| Argentina | 6 | 54.46 | 3.60 | 39 | 120.95 | 0.46 | 45 |
| Nigeria | 16 | 20.66 | 1.37 | 32 | 167.81 | 0.64 | 48 |
| Bolivia | 36 | 9.74 | 0.64 | 18 | 231.14 | 0.88 | 54 |
| Australia | 13 | 24.72 | 1.64 | 41 | 111.92 | 0.43 | 54 |
| South Africa | 28 | 12.59 | 0.83 | 27 | 173.49 | 0.66 | 55 |
| Viet Nam | 45 | 6.06 | 0.40 | 11 | 382.60 | 1.46 | 56 |
| Iran | 51 | 5.19 | 0.34 | 6 | 1091.35 | 4.16 | 57 |
| Ethiopia | 5 | 63.28 | 4.19 | 53 | 64.46 | 0.25 | 58 |
| Canada | 31 | 11.50 | 0.76 | 29 | 171.40 | 0.65 | 60 |
| Venezuela | 23 | 16.05 | 1.06 | 38 | 123.17 | 0.47 | 61 |
| UK & Northern Ireland | 37 | 9.74 | 0.64 | 26 | 176.00 | 0.67 | 63 |
| Germany[c] | 30 | 11.64 | 0.77 | 34 | 160.00 | 0.61 | 64 |
| Sudan | 9 | 31.49 | 2.08 | 58 | 50.02 | 0.19 | 67 |
| Poland | 44 | 6.26 | 0.41 | 24 | 183.12 | 0.70 | 68 |
| Kenya | 15 | 20.90 | 1.38 | 54 | 56.66 | 0.22 | 69 |
| Thailand | 56 | 4.60 | 0.30 | 15 | 281.49 | 1.07 | 71 |
| Japan | 61 | 3.84 | 0.25 | 13 | 323.15 | 1.23 | 74 |
| Uzbekistan | 27 | 12.95 | 0.86 | 50 | 73.28 | 0.28 | 77 |
| Italy[3] | 43 | 6.38 | 0.42 | 35 | 146.95 | 0.56 | 78 |
| Spain[3] | 41 | 6.60 | 0.44 | 37 | 137.50 | 0.52 | 78 |
| Peru | 48 | 5.60 | 0.37 | 31 | 168.17 | 0.64 | 79 |
| United Republic of Tanzania | 11 | 27.82 | 1.84 | 70 | 38.47 | 0.15 | 81 |
| Egypt | 53 | 4.90 | 0.32 | 30 | 169.08 | 0.64 | 83 |
| Morocco | 68 | 3.33 | 0.22 | 19 | 208.63 | 0.80 | 87 |
| Ukraine | 67 | 3.33 | 0.22 | 21 | 191.97 | 0.73 | 88 |
| Nepal | 40 | 7.39 | 0.49 | 48 | 75.71 | 0.29 | 88 |
| Mali | 29 | 12.11 | 0.80 | 59 | 49.62 | 0.19 | 88 |
| Ecuador | 57 | 4.31 | 0.28 | 33 | 161.07 | 0.61 | 90 |
| Republic of Korea | 66 | 3.65 | 0.24 | 28 | 172.92 | 0.66 | 94 |
| Dominican Republic | 70 | 3.08 | 0.20 | 25 | 181.74 | 0.69 | 95 |
| Uganda | 22 | 16.33 | 1.08 | 73 | 37.04 | 0.14 | 95 |
| Philippines | 74 | 2.54 | 0.17 | 23 | 186.37 | 0.71 | 97 |
| Cameroon | 46 | 5.77 | 0.38 | 55 | 55.12 | 0.21 | 101 |
| Kazakhstan | 39 | 7.44 | 0.49 | 63 | 43.42 | 0.17 | 102 |
| Madagascar | 35 | 9.98 | 0.66 | 67 | 39.92 | 0.15 | 102 |
| Burkina Faso | 34 | 10.00 | 0.66 | 69 | 38.87 | 0.15 | 103 |
| Netherlands[3] | 63 | 3.72 | 0.25 | 42 | 105.18 | 0.40 | 105 |

| | | | | | | | |
|---|---|---|---|---|---|---|---|
| Chile | 69 | 3.11 | 0.21 | 40 | 114.44 | 0.44 | 109 |
| Senegal | 65 | 3.67 | 0.24 | 46 | 81.42 | 0.31 | 111 |
| Paraguay | 25 | 13.80 | 0.91 | 87 | 24.75 | 0.09 | 112 |
| Uruguay | 32 | 11.40 | 0.75 | 81 | 31.37 | 0.12 | 113 |
| Angola | 52 | 5.09 | 0.34 | 62 | 44.37 | 0.17 | 114 |
| Guinea | 38 | 7.93 | 0.53 | 76 | 36.18 | 0.14 | 114 |
| New Zealand | 33 | 10.15 | 0.67 | 88 | 24.72 | 0.09 | 121 |
| Niger | 24 | 15.23 | 1.01 | 97 | 19.70 | 0.08 | 121 |
| Algeria | 88 | 1.78 | 0.12 | 36 | 138.58 | 0.53 | 124 |
| Belarus | 58 | 4.29 | 0.28 | 66 | 41.06 | 0.16 | 124 |
| Zambia | 64 | 3.68 | 0.24 | 64 | 41.88 | 0.16 | 128 |
| Ghana | 84 | 1.94 | 0.13 | 45 | 89.21 | 0.34 | 129 |
| Malaysia | 114 | 0.68 | 0.05 | 16 | 275.80 | 1.05 | 130 |
| Honduras | 72 | 2.90 | 0.19 | 61 | 47.20 | 0.18 | 133 |
| Guatemala | 60 | 4.01 | 0.27 | 74 | 36.93 | 0.14 | 134 |
| Romania | 86 | 1.92 | 0.13 | 49 | 73.99 | 0.28 | 135 |
| Côte d'Ivoire | 90 | 1.69 | 0.11 | 47 | 76.74 | 0.29 | 137 |
| South Sudan | 26 | 13.14 | 0.87 | 113 | 15.00 | 0.06 | 139 |
| Iraq | 83 | 1.98 | 0.13 | 57 | 50.07 | 0.19 | 140 |
| Nicaragua | 50 | 5.22 | 0.35 | 90 | 24.25 | 0.09 | 140 |
| Saudi Arabia | 121 | 0.57 | 0.04 | 20 | 196.99 | 0.75 | 141 |
| Chad | 10 | 30.61 | 2.03 | 134 | 6.31 | 0.02 | 144 |
| Lao People's Democratic Republic | 81 | 2.09 | 0.14 | 65 | 41.39 | 0.16 | 146 |
| Cuba | 62 | 3.75 | 0.25 | 85 | 26.70 | 0.10 | 147 |
| Yemen | 98 | 1.41 | 0.09 | 52 | 69.06 | 0.26 | 150 |
| Ireland[3] | 42 | 6.56 | 0.43 | 111 | 15.50 | 0.06 | 153 |
| Belgium[2] | 77 | 2.37 | 0.16 | 78 | 32.33 | 0.12 | 155 |
| Azerbaijan | 76 | 2.48 | 0.16 | 79 | 32.23 | 0.12 | 155 |
| Tunisia | 115 | 0.65 | 0.04 | 44 | 92.66 | 0.35 | 159 |
| Afghanistan | 47 | 5.65 | 0.37 | 115 | 13.89 | 0.05 | 162 |
| Benin | 75 | 2.50 | 0.17 | 93 | 21.98 | 0.08 | 168 |
| Zimbabwe | 49 | 5.52 | 0.37 | 129 | 8.75 | 0.03 | 178 |
| Panama | 96 | 1.50 | 0.10 | 84 | 28.14 | 0.11 | 180 |
| Israel | 122 | 0.55 | 0.04 | 60 | 49.04 | 0.19 | 182 |
| Turkmenistan | 79 | 2.27 | 0.15 | 103 | 16.91 | 0.06 | 182 |
| Costa Rica | 97 | 1.47 | 0.10 | 86 | 25.81 | 0.10 | 183 |
| Hungary[2] | 105 | 0.91 | 0.06 | 80 | 31.84 | 0.12 | 185 |
| Central African Republic | 54 | 4.76 | 0.31 | 131 | 7.93 | 0.03 | 185 |
| Denmark | 95 | 1.50 | 0.10 | 92 | 22.46 | 0.09 | 187 |
| Malawi | 89 | 1.74 | 0.11 | 98 | 18.78 | 0.07 | 187 |
| Taiwan | 145 | 0.15 | 0.01 | 43 | 98.67 | 0.38 | 188 |
| Mozambique | 82 | 2.09 | 0.14 | 106 | 16.70 | 0.06 | 188 |
| Cambodia | 73 | 2.85 | 0.19 | 116 | 13.26 | 0.05 | 189 |
| Austria[c] | 87 | 1.88 | 0.12 | 104 | 16.74 | 0.06 | 191 |
| Czechia | 100 | 1.37 | 0.09 | 94 | 21.84 | 0.08 | 194 |
| Greece[2] | 123 | 0.53 | 0.04 | 72 | 37.30 | 0.14 | 195 |
| Sri Lanka | 104 | 1.09 | 0.07 | 96 | 20.41 | 0.08 | 200 |
| Democratic Republic of the Congo | 102 | 1.21 | 0.08 | 99 | 18.56 | 0.07 | 201 |
| Lebanon | 153 | 0.09 | 0.01 | 51 | 73.20 | 0.28 | 204 |
| Togo | 128 | 0.46 | 0.03 | 77 | 33.24 | 0.13 | 205 |
| Tajikistan | 78 | 2.36 | 0.16 | 128 | 9.04 | 0.03 | 206 |
| Somalia | 59 | 4.23 | 0.28 | 148 | 3.72 | 0.01 | 207 |
| Sierra Leone | 118 | 0.58 | 0.04 | 91 | 23.73 | 0.09 | 209 |
| Syrian Arab Republic | 111 | 0.79 | 0.05 | 100 | 18.50 | 0.07 | 211 |

| | | | | | | | |
|---|---|---|---|---|---|---|---|
| El Salvador | 110 | 0.84 | 0.06 | 101 | 17.97 | 0.07 | 211 |
| Switzerland | 93 | 1.52 | 0.10 | 119 | 11.83 | 0.05 | 212 |
| Namibia | 71 | 2.93 | 0.19 | 142 | 4.35 | 0.02 | 213 |
| Republic of Moldova | 146 | 0.14 | 0.01 | 68 | 39.86 | 0.15 | 214 |
| Portugal[3] | 92 | 1.67 | 0.11 | 122 | 10.76 | 0.04 | 214 |
| Libya | 140 | 0.23 | 0.02 | 75 | 36.55 | 0.14 | 215 |
| Norway | 108 | 0.87 | 0.06 | 108 | 15.85 | 0.06 | 216 |
| Serbia | 106 | 0.90 | 0.06 | 112 | 15.42 | 0.06 | 218 |
| Bulgaria | 124 | 0.53 | 0.03 | 95 | 20.79 | 0.08 | 219 |
| Mongolia | 55 | 4.75 | 0.31 | 167 | 0.89 | 0.00 | 222 |
| Kuwait | 167 | 0.03 | 0.00 | 56 | 54.64 | 0.21 | 223 |
| Mauritania | 85 | 1.93 | 0.13 | 140 | 4.68 | 0.02 | 225 |
| Sweden[2] | 99 | 1.40 | 0.09 | 127 | 9.20 | 0.04 | 226 |
| Democratic People's Republic of Korea | 119 | 0.58 | 0.04 | 109 | 15.77 | 0.06 | 228 |
| Haiti | 94 | 1.51 | 0.10 | 135 | 5.84 | 0.02 | 229 |
| Kyrgyzstan | 91 | 1.68 | 0.11 | 139 | 4.82 | 0.02 | 230 |
| Guyana | 151 | 0.10 | 0.01 | 83 | 29.17 | 0.11 | 234 |
| Finland | 109 | 0.84 | 0.06 | 125 | 9.51 | 0.04 | 234 |
| Trinidad and Tobago | 165 | 0.04 | 0.00 | 71 | 38.44 | 0.15 | 236 |
| Lithuania | 116 | 0.63 | 0.04 | 120 | 11.56 | 0.04 | 236 |
| Bosnia and Herzegovina | 130 | 0.43 | 0.03 | 107 | 16.67 | 0.06 | 237 |
| Georgia | 107 | 0.88 | 0.06 | 130 | 7.94 | 0.03 | 237 |
| Rwanda | 101 | 1.33 | 0.09 | 136 | 5.57 | 0.02 | 237 |
| Jordan | 157 | 0.08 | 0.01 | 82 | 29.73 | 0.11 | 239 |
| United Arab Emirates | 150 | 0.11 | 0.01 | 89 | 24.67 | 0.09 | 239 |
| Eritrea | 80 | 2.12 | 0.14 | 162 | 1.13 | 0.00 | 242 |
| Puerto Rico | 136 | 0.36 | 0.02 | 110 | 15.57 | 0.06 | 246 |
| Slovakia | 129 | 0.43 | 0.03 | 117 | 12.85 | 0.05 | 246 |
| Croatia | 131 | 0.42 | 0.03 | 118 | 12.16 | 0.05 | 249 |
| Jamaica | 147 | 0.12 | 0.01 | 114 | 14.35 | 0.05 | 261 |
| Eswatini | 117 | 0.62 | 0.04 | 146 | 3.96 | 0.02 | 263 |
| Albania | 132 | 0.42 | 0.03 | 132 | 7.27 | 0.03 | 264 |
| Armenia | 120 | 0.57 | 0.04 | 144 | 4.15 | 0.02 | 264 |
| Burundi | 112 | 0.76 | 0.05 | 153 | 2.73 | 0.01 | 265 |
| Guinea-Bissau | 113 | 0.75 | 0.05 | 155 | 2.11 | 0.01 | 268 |
| Oman | 133 | 0.40 | 0.03 | 138 | 4.91 | 0.02 | 271 |
| Slovenia | 126 | 0.48 | 0.03 | 145 | 4.03 | 0.02 | 271 |
| Botswana | 103 | 1.10 | 0.07 | 173 | 0.50 | 0.00 | 276 |
| Mauritius | 181 | 0.00 | 0.00 | 105 | 16.73 | 0.06 | 286 |
| Qatar | 162 | 0.04 | 0.00 | 124 | 9.71 | 0.04 | 286 |
| Fiji | 149 | 0.11 | 0.01 | 137 | 5.47 | 0.02 | 286 |
| Congo | 135 | 0.37 | 0.02 | 151 | 3.05 | 0.01 | 286 |
| Gambia | 127 | 0.47 | 0.03 | 159 | 1.48 | 0.01 | 286 |
| Palestine | 166 | 0.03 | 0.00 | 121 | 11.23 | 0.04 | 287 |
| Liberia | 161 | 0.05 | 0.00 | 126 | 9.23 | 0.04 | 287 |
| Latvia | 134 | 0.40 | 0.03 | 154 | 2.34 | 0.01 | 288 |
| Brunei Darussalam | 188 | 0.00 | 0.00 | 102 | 16.98 | 0.06 | 290 |
| Papua New Guinea | 152 | 0.09 | 0.01 | 141 | 4.66 | 0.02 | 293 |
| Estonia | 139 | 0.25 | 0.02 | 156 | 2.08 | 0.01 | 295 |
| Suriname | 164 | 0.04 | 0.00 | 133 | 6.35 | 0.02 | 297 |
| Bhutan | 137 | 0.30 | 0.02 | 161 | 1.30 | 0.00 | 298 |
| Bahrain | 176 | 0.01 | 0.00 | 123 | 9.84 | 0.04 | 299 |
| North Macedonia | 141 | 0.22 | 0.01 | 158 | 1.56 | 0.01 | 299 |
| Lesotho | 125 | 0.51 | 0.03 | 176 | 0.42 | 0.00 | 301 |

| | | | | | | | |
|---|---|---|---|---|---|---|---|
| Belize | 148 | 0.12 | 0.01 | 157 | 1.64 | 0.01 | 305 |
| Timor-Leste | 142 | 0.21 | 0.01 | 166 | 0.90 | 0.00 | 308 |
| Vanuatu | 144 | 0.18 | 0.01 | 165 | 0.98 | 0.00 | 309 |
| Cyprus | 158 | 0.07 | 0.00 | 152 | 2.76 | 0.01 | 310 |
| Gabon | 163 | 0.04 | 0.00 | 149 | 3.19 | 0.01 | 312 |
| Barbados | 174 | 0.01 | 0.00 | 143 | 4.20 | 0.02 | 317 |
| Montenegro | 155 | 0.08 | 0.01 | 170 | 0.69 | 0.00 | 325 |
| New Caledonia | 154 | 0.08 | 0.01 | 174 | 0.48 | 0.00 | 328 |
| Comoros | 160 | 0.05 | 0.00 | 171 | 0.55 | 0.00 | 331 |
| Samoa | 159 | 0.06 | 0.00 | 172 | 0.51 | 0.00 | 331 |
| Luxembourg | 143 | 0.19 | 0.01 | 188 | 0.13 | 0.00 | 331 |
| Cabo Verde | 168 | 0.03 | 0.00 | 164 | 1.00 | 0.00 | 332 |
| Djibouti | 138 | 0.30 | 0.02 | 196 | 0.00 | 0.00 | 334 |
| Malta[c] | 172 | 0.01 | 0.00 | 163 | 1.00 | 0.00 | 335 |
| Singapore | 190 | 0.00 | 0.00 | 147 | 3.79 | 0.01 | 337 |
| Bahamas | 187 | 0.00 | 0.00 | 150 | 3.05 | 0.01 | 337 |
| Iceland | 156 | 0.08 | 0.01 | 184 | 0.23 | 0.00 | 340 |
| China, Hong Kong SAR | 185 | 0.00 | 0.00 | 160 | 1.38 | 0.01 | 345 |
| Saint Lucia | 175 | 0.01 | 0.00 | 175 | 0.47 | 0.00 | 350 |
| Tonga | 173 | 0.01 | 0.00 | 178 | 0.35 | 0.00 | 351 |
| Solomon Islands | 170 | 0.02 | 0.00 | 183 | 0.25 | 0.00 | 353 |
| Equatorial Guinea | 178 | 0.01 | 0.00 | 177 | 0.38 | 0.00 | 355 |
| Micronesia | 169 | 0.02 | 0.00 | 186 | 0.19 | 0.00 | 355 |
| Dominica | 171 | 0.01 | 0.00 | 185 | 0.20 | 0.00 | 356 |
| French Polynesia | 177 | 0.01 | 0.00 | 180 | 0.33 | 0.00 | 357 |
| Grenada | 179 | 0.00 | 0.00 | 181 | 0.28 | 0.00 | 360 |
| China, Macao SAR | 193 | 0.00 | 0.00 | 168 | 0.79 | 0.00 | 361 |
| Kiribati | 194 | 0.00 | 0.00 | 169 | 0.70 | 0.00 | 363 |
| Saint Vincent and the Grenadines | 182 | 0.00 | 0.00 | 182 | 0.25 | 0.00 | 364 |
| Sao Tome and Principe | 186 | 0.00 | 0.00 | 179 | 0.34 | 0.00 | 365 |
| Antigua and Barbuda | 180 | 0.00 | 0.00 | 187 | 0.16 | 0.00 | 367 |
| Saint Kitts and Nevis | 183 | 0.00 | 0.00 | 189 | 0.10 | 0.00 | 372 |
| Seychelles | 189 | 0.00 | 0.00 | 191 | 0.05 | 0.00 | 380 |
| Faroe Islands | 184 | 0.00 | 0.00 | 197 | 0.00 | 0.00 | 381 |
| Niue | 192 | 0.00 | 0.00 | 192 | 0.02 | 0.00 | 384 |
| Cook Islands | 191 | 0.00 | 0.00 | 193 | 0.02 | 0.00 | 384 |
| Tuvalu | 195 | 0.00 | 0.00 | 190 | 0.06 | 0.00 | 385 |
| Tokelau | 196 | 0.00 | 0.00 | 194 | 0.01 | 0.00 | 390 |
| Nauru | 197 | 0.00 | 0.00 | 195 | 0.01 | 0.00 | 392 |

[1]Livestock density data is for 2019 unless indicated otherwise.
[2]Chicken density data is for 2018.
[3]Chicken density data is for 2017.

## References

1. Alberta Government. 2011 Census of agriculture for Alberta. *Alberta Agriculture and Rural Development* **2014**, *https://www1.agric.gov.ab.ca/$Department/deptdocs.nsf/all/agdex4091/$FILE/852-6.pdf* (Accessed 4 November 2022).
2. Alberta Government. 2011 Municipal affairs population list. **2011**, *https://open.alberta.ca/opendata/alberta-municipal-affairs-population-list* (Accessed 4 November 2022).
3. Government of Canada. Agricultural operation characteristics Canada. *Statistics Canada* **2016**, *https://www150.statcan.gc.ca/n1/pub/95-634-x/2017001/article/54899-eng.htm* (Accessed 4 November 2022).
4. Public Health Agency of Canada. Notifiable diseases online. **2018**, *http://diseases.canada.ca/notifiable/* (Accessed 4 November 2022).
5. Inglis, G.D.; Boras, V.F.; Webb, A.L.; Suttorp, V.V.; Hodgkinson, P.; Taboada, E.N. Enhanced microbiological surveillance reveals that temporal case clusters contribute to the high rates of campylobacteriosis in a model agroecosystem. *Int J Med Microbiol* **2019**, *309*, 232-244, doi:10.1016/j.ijmm.2019.04.003.
6. Alberta Health and Wellness. Health trends in Alberta. **2007**, *https://open.alberta.ca/dataset/a4731b3e-9848-42a2-be69-eeae8f9bcf41/resource/9d244198-8fcc-43ee-bfee-f859ec7e1545/download/trends-2007-health.pdf (Accessed 4 November 2022)*.
7. Food and Agricultural Organization of the United Nations. Live animals. **2019**, *http://www.fao.org/faostat/en/#data/QA* (Accessed 4 November 2022).
